# Silencing of D-Lactate Dehydrogenase Impedes Glyoxalase System and Leads to Methylglyoxal Accumulation and Growth Inhibition in Rice

**DOI:** 10.3389/fpls.2017.02071

**Published:** 2017-12-05

**Authors:** Baoguang An, Jie Lan, Xiaolong Deng, Silan Chen, Chao Ouyang, Huiyun Shi, Jing Yang, Yangsheng Li

**Affiliations:** ^1^State Key Laboratory of Hybrid Rice, Key Laboratory for Research and Utilization of Heterosis in Indica Rice, Ministry of Agriculture, The Yangtze River Valley Hybrid Rice Collaboration Innovation Center, College of Life Sciences, Wuhan University, Wuhan, China; ^2^Hainan Bolian Rice Gene Technology Co., Ltd., Haikou, China

**Keywords:** alternative splicing, D-lactate dehydrogenase, growth inhibition, GSH, methylglyoxal, glyoxalase system, aldo-keto reductase, photorespiration

## Abstract

D-Lactate is oxidized by two classes of D-lactate dehydrogenase (D-LDH), namely, NAD-dependent and NAD-independent D-LDHs. Little is known about the characteristics and biological functions of D-LDHs in rice. In this study, a functional NAD-independent D-LDH (LOC_Os07g06890) was identified in rice, as a result of alternative splicing events. Characterization of the expression profile, subcellular localization, and enzymatic properties of the functional OsD-LDH revealed that it is a mitochondrial cytochrome-*c*-dependent D-LDH with high affinity and catalytic efficiency. Functional analysis of *OsD-LDH* RNAi transgenic rice demonstrated that OsD-LDH participates in methylglyoxal metabolism by affecting the activity of the glyoxalase system and aldo-keto reductases. Under methylglyoxal treatment, silencing of OsD-LDH in rice resulted in the accumulation of methylglyoxal and D-lactate, the decrease of reduced glutathione in leaves, and ultimately severe growth inhibition. Moreover, the detached leaves of *OsD-LDH* RNAi plants were more sensitive to salt stress. However, the silencing of OsD-LDH did not affect the growth under photorespiration conditions. Our results provide new insights into the role of NAD-independent D-LDHs in rice.

## Introduction

Methylglyoxal (MG), a highly reactive α-oxoaldehyde, is a byproduct of glycolysis, lipid peroxidation, and the oxidative degradation of glucose and glycated proteins (Kalapos, 1999; Hoque et al., 2012b). Accumulated MG can interact with nucleic acids and proteins, resulting in the disruption of cellular functions and inhibition of cellular proliferation (Murata-Kamiya and Kamiya, 2001; Thornalley, 2006). In plants, large amounts of MG, accumulated under a variety of stress conditions (Yadav et al., 2008), can inhibit seed germination, root elongation, and stomatal opening (Hoque et al., 2012a,b, 2016). MG is mainly degraded by the glyoxalase system (Kalapos, 1999; Yadav et al., 2005), through which it is converted to *S*-lactoylglutathione (SLG) by glyoxalase I (Gly I) in the presence of its cofactor reduced glutathione (GSH). The resulting SLG is split into GSH and D-lactate by glyoxalase II (Gly II) (Racker, 1951). Previous studies have shown that the glyoxalase system regulates plant cell proliferation and differentiation (Deswal et al., 1993) and participates in response to abiotic or biotic stressors and hormones (Khan et al., 2005; Saxena et al., 2005; Du et al., 2010; Hossain et al., 2012; Mostofa and Fujita, 2013). There are eleven Gly I and three Gly II genes in the rice genome (Mustafiz et al., 2011). One of the Gly I proteins, OsGLYI-11.2, was previously characterized as an efficient Ni^2+^-dependent glyoxalase that could be induced by methylglyoxal (Mustafiz et al., 2014). Two of the Gly II proteins, Gly II-2 and Gly II-3, were confirmed as efficient glyoxalase II proteins that contribute to MG scavenging and abiotic stress tolerance (Singla-Pareek, 2003; Ghosh et al., 2014). The overexpression of Gly I, Gly II, or both contributes to improved adaptation to various abiotic stresses in a range of plants (Reddy and Sopory, 1999; Singla-Pareek, 2003; Singla-Pareek et al., 2006, 2008; Bhomkar et al., 2008; Lin et al., 2010; Álvarez Viveros et al., 2013; Ghosh et al., 2014; Mustafiz et al., 2014). Recently, a functional glyoxalase III enzyme was identified in rice, which is able to convert MG into D-lactate in a single step independent of glutathione, although its efficiency was relatively low compared with the conventional Gly I/II enzymes (Ghosh et al., 2016).

Whether the detoxification of MG occurs through the major pathway, the Gly I/II system, or through the minor pathway, the Gly III system, the end product is D-lactate. D-Lactate is one of the two optical isomers of lactate. Because of its cytotoxicity, lactate can modify gene expression, energy production, cancer development, and cell acidosis (Felle, 2005; Hirschhaeuser et al., 2011; Latham et al., 2012; Ling et al., 2012). Thus, the conversion of lactate to pyruvate is necessary and this process is catalyzed by various evolutionarily unrelated families of enzymes, including NAD-dependent L-lactate dehydrogenase (L-LDH) (EC 1.1.1.27) and D-lactate dehydrogenase (D-LDH) (EC 1.1.1.28), and NAD-independent L-LDH (EC 1.1.2.3) and D-LDH (EC 1.1.2.4) (Cristescu et al., 2008). After being detected in some microbes (Brockman and Wood, 1975; Ogata et al., 1981; Horikiri et al., 2004; Ma et al., 2007), cytochrome *c* (Cyt *c*)-dependent D-LDHs belonging to the NAD-independent LDH family were reported to catalyze the oxidation of D-lactate to pyruvate in yeast mitochondria (Lodi and Ferrero, 1993; Lodi et al., 1994). In plants, the activity of Cyt-*c*-dependent D-LDH was first detected in the mitochondria of *Helianthus tuberosus* (Atlante et al., 2005). Subsequently, a mitochondrial Cyt-*c*-dependent D-LDH that catalyzes the oxidation of D-lactate to pyruvate in *Arabidopsis thaliana* was characterized (Engqvist et al., 2009). The same protein was also found to be a glycolate dehydrogenase (GDH) involved in photorespiration (Bari et al., 2004; Niessen et al., 2007).

A candidate locus for a D-lactate dehydrogenase or GDH has been annotated in the rice genome (LOC_Os07g06890); however, the predicted protein is truncated. Whether there is a functional D-lactate/GDH in rice has so far remained unclear. If so, the structure, kinetics, and biological functions need to be elucidated. Hence, we isolated the mRNA for this locus and thereby unexpectedly identified a full-length functional D-lactate dehydrogenase as a result of alternative splicing. RNAi transgenic rice plants were generated to investigate the function of OsD-LDH *in vivo*, and the potential roles of OsD-LDH in rice are discussed.

## Materials and Methods

### Plant Materials and Growth Conditions

Rice plants (*Oryza sativa* L. ssp. *indica* ‘93-11’ (93-11 in short) and *O. sativa* L. ssp. *japonica* ‘Zhonghua 11’ (ZH11 in short)) were grown in a greenhouse with a 16/8-h light/dark photoperiod at 30/20°C at Wuhan University or under field conditions in Wuhan during summer in 2011–2014.

### BLAST Search and Cloning of *OsD-LDH* cDNA

The Rice RefSeq RNA Database was searched with the protein sequences of AtD-LDH (At5g06580) and ScDLD1 (CAA46852) using the TBLASTN algorithm. The cDNAs of *OsD-LDH* were isolated from young leaves of rice plants (93-11) by RT-PCR using gene-specific primers encompassing the 5′ translation start codon and 3′ untranslated region, 5′-GGACTAGTATGGCCACCGCCG-3′ and 5′-GCGTCGACTCAAATGCAGACTTGAGGTG-3′, which contained an additional *Spe*I site and *Sal*I site, respectively. PCR fragments were ligated into the plasmid pBluescript II SK (Fermentas, United States), and the generated plasmids, designated pBSK-OsD-LDH, were sequenced. The truncated *OsD-LDH* and the full-length *OsD-LDH* were designated pBSK-OsD-LDH-Tr and pBSK-OsD-LDH-FL, respectively.

### Expression and Purification of Recombinant OsD-LDH

For protein expression, the truncated *OsD-LDH* (*OsD-LDH-Tr*) and the full-length *OsD-LDH* (*OsD-LDH-FL*) were amplified by PCR using pBSK-OsD-LDH-Tr and pBSK-OsD-LDH-FL as the templates, respectively, with the gene-specific primers 5′-GCGTCGACTCATGGCCACCGCCG-3′ and 5′-GAGCGGCCGCTCAAATGCAGACTTGAGGTG-3′, which contained an additional *Sal*I site and *Not*I site, respectively. The PCR fragments were ligated into plasmid pGEX-6P-1 (GE, United States), and the generated constructs, designated pGEX-OsD-LDH-Tr and pGEX-OsD-LDH-FL, respectively, were confirmed by sequencing. The constructs pGEX-OsD-LDH-Tr and pGEX-OsD-LDH-FL were used to generate fusion proteins with an N-terminal GST tag, to facilitate purification of the recombinant proteins. One-liter cultures of *Escherichia coli* BL21 (DE3) cells transformed with the constructs were grown in LB medium at 37°C and 220 rpm in the presence of 60 μg mL^-1^ ampicillin until the cultures reached an OD_600_ of 0.5 to 0.7. The expression of recombinant OsD-LDH-Tr and OsD-LDH-FL was induced by addition of 0.5 mM IPTG, and the cultures were grown at 18°C for another 4–6 h. The cells were then harvested by centrifugation at 12,000 × *g* for 3 min, washed once with 1 × PBS, and resuspended in the same buffer to obtain a 10–15% (v/v) cell suspension with 0.1% (v/v) Triton X-100. After lysis of the cells by sonication over ice, the extracts were centrifuged at 18,000 × *g* and 4°C for 30 min to remove debris. The supernatants were used for protein purification with Glutathione Sepharose 4B (GE, United States) according to the manufacturer’s instructions. Next, the highly purified recombinant OsD-LDH-Tr and OsD-LDH-FL proteins were confirmed by western blotting using anti-GST antibody. The purified proteins were used immediately for enzymatic activity measurements or stored at -20°C.

### Enzymatic Assays

The activities of OsD-LDH were determined spectrophotometrically with a Tecan Infinite M200 PRO microplate reader. The reaction mixture contained 50 mM K_3_PO_4_ buffer (pH 5.0–10), the substrates with a series of concentrations, the purified proteins (0.5–10 μg) and electron acceptors including 200 μM cytochrome *c* (Cyt *c*), 250 μM NAD(P)^+^/NAD(P)H, or 3 mM phenazine methosulfate and 200 μM 2,6-dichlorophenolindophenol (PMS-DCIP), respectively, in a final volume of 0.2 mL. The oxidation of substrates using Cyt *c*, PMS-DCIP and NAD(P)^+^/NAD(P)H as electron acceptors was measured at 550, 600, and 340 nm, respectively. Using PMS-DCIP and NAD(P)^+^/NAD(P)H as electron acceptors, the absorptions were equivalent to the substrates oxidized, and using Cyt *c* as electron acceptors, the absorption was equivalent to two times the substrates oxidized.

The substrate specificity of the enzymes was screened among a series of substrates at the concentration of 10 mM, including D-2-hydroxybutyrate, D-lactate, L-lactate, glycerol, glycolate and 3-hydroxybutyrate, together with Cyt *c* at pH 8.35 and 25°C. The electron acceptors were screened among Cyt *c* and NAD(P)^+^/NAD(P)H with D-lactate or pyruvate as substrates at the concentration of 10 mM, at pH 8.35 and 25°C. The optimal pH was assayed with PMS-DCIP and 200 μM D-lactate at 25°C. The optimal temperature was assayed with PMS-DCIP and 200 μM D-lactate at pH 8.35. The kinetic parameters for D-lactate and D-2-HB were determined using a series of substrate concentrations with Cyt *c* or PMS-DCIP as electron acceptors, respectively, at pH 8.35 and 30°C.

All of the parameters were calculated using at least two independent enzyme batches each consisting of at least triplicate determinations.

### Preparation of OsD-LDH-GFP Fusion Constructs and Subcellular Localization

To determine the subcellular localization of OsD-LDH in rice, the entire open reading frame without the stop codon was amplified by PCR from full-length *OsD-LDH* using the primers 5′-CTAGATCTGATGGCCACCGCCG-3′ and 5′-GGACTAGTAATGCAGACTTGAGGTGGA-3′, containing an additional *Bgl*II site and *Spe*I site, respectively. The PCR fragments were ligated into the binary vector pCAMBIA1302 with GFP fused in-frame to its C terminus, and the generated construct was designated pCDLDH-GFP.

To determine the location and length of the targeting peptide, a series of different lengths of the OsD-LDH coding sequence were fused in-frame to the N terminus of the GFP of pCAMBIA1302, which contained the first 60, 120, or 246 bp of the coding sequence. The common forward primer was 5′-CGAGCCATGGCCACCGCCG-3′ with an additional *Nco*I site. The associated reverse primers were 5′-GGACTAGTGGGGAGGAGCGGGC-3′, 5′-GGACTAGTGGTTTGGGAGTGGGAGTG-3′, and 5′-GGACTAGTCCGGTGGTCGATGCC-3′ with an additional *Spe*I site. The generated constructs were designated pCDLDH_20_-GFP, pCDLDH_40_-GFP, and pCDLDH_82_-GFP, respectively. The 1444-bp region of the 3′ end of the full-length *OsD-LDH* coding sequence without the first 239 bp and stop codon was amplified by PCR using the primers 5′-CTAGATCTCCACCGGGTCGGG-3′ and 5′-GGACTAGTAATGCAGACTTGAGGTGGA-3′ containing an additional *Bgl*II site and *Spe*I site, respectively. The PCR fragments were ligated into pCAMBIA1302 with GFP fused in-frame to the C terminus, and the generated construct was designated pCDLDH_C′_-GFP. All of the constructs were confirmed by sequencing and transformed into rice plants (ZH11) by *Agrobacterium*-mediated transformation as previously described (Hiei et al., 1994). The expression of all of the generated GFP fusion proteins was driven by the CaMV 35S promoter.

Protoplast preparation was performed according to a previous study (Bari et al., 2004) with modifications. Ten-day-old etiolated transgenic rice seedlings were cut into small sections (approximately 0.5 mm in length) and incubated in protoplast isolation buffer [0.6 M mannitol, 10 mM MES buffer (pH 5.7), 1 mM CaCl_2_, 0.1% BSA (w/v), 0.04% β-mercaptoethanol (v/v), 1.5% cellulose R-10 (w/v), and 0.75% macerozyme R-10 (w/v; Yakult Honsha), supplemented with 50 μg mL^-1^ carbenicillin) at 28°C for 4 h to release the protoplast cells. The supernatant containing the protoplasts was carefully centrifuged at 100 × *g* for 5 min, resuspended in W5 buffer (pH 5.8, 154 mM NaCl, 125 mM CaCl_2_, 5 mM KCl, 5 mM glucose, and 2 mM MES), and then stained with MitoTracker Red (Invitrogen) according to the manufacturer’s instructions. Images of the protoplasts were obtained using an Olympus FV1000 confocal microscope. The GFP and MitoTracker Red fluorescence signals were excited with 488 and 559 nm laser lines and detected at 500–545 nm and 600–670 nm, respectively.

### Tissue Expression Analysis

RNA was prepared from various tissues of rice plants (93-11) using TRIzol (Invitrogen, United States). First-strand cDNA was synthesized from DNase I-treated total RNA using M-MLV reverse transcriptase (Promega, United States) according to the manufacturer’s instructions. Quantitative real-time PCR (qRT-PCR) was performed using a Bio-Rad CFX96^TM^ Real-Time PCR system. The expression of *OsD-LDH* (primers: 5′-AACTGATTCCACCTCAAGTCTGC-3′ and 5′-GAGTCTTCGCCGTTCAATGC-3′) in various tissue samples was determined using the comparative Ct method and normalized to the expression of the internal control genes using the multiple reference genes strategy. The multiple reference genes are Actin1 (primers: 5′-AGCATGAAGATCAAGGTGGTC-3′ and 5′-GCCTTGGCAATCCACATC-3′), UBQ5 (primers: 5′-GGAAGGAGGAGGAAATCGAACT-3′ and 5′-TCTTCACAGAGGTGATGCTAAGGT-3′) and ADP-ribosylation factor (ARF) (primers: 5′-GCTTACGGTGCCTGACTTTTG-3′ and 5′-GGGATAAACTGGTAAGGATATTGGG-3′).

### Preparation of OsD-LDH RNAi Constructs and Transformation

For functional analysis of OsD-LDH, a 493-bp fragment of *OsD-LDH*, including the complete exon 12, 13, 14, and 15 and part of the exons 11 and 16, was amplified by PCR from *OsD-LDH* using primers 5′-CCGCTCGAGGGATCCTTGCAACAATGCTTTCTGG-3′ and 5′-GGGGTACCATCGATATGAAATGGTTTAGCCTCTCC-3′, which contained an additional *Xho*I/*BamH*I site and *Kpn*I/*Cla*I site, respectively. The PCR fragments were ligated on both sides of the Pdk Intron (Wesley et al., 2001) in opposite directions, and the generated fragments were positioned downstream of the maize ubiquitin promoter in the binary vector pCAMBIA1301 and introduced into rice (ZH11) by *Agrobacterium*-mediated transformation as previously described (Hiei et al., 1994). The empty vector pCAMBIA1301 was also transformed into rice as a negative control. The expression of *OsD-LDH* in young leaves of T1 progeny of *OsD-LDH* RNAi transgenic rice plants was evaluated by qRT-PCR using the primers described above (Tissue Expression Analysis).

### MG, Illumination and NaCl Treatment

Wild type (ZH11) plants were grown on ½ Murashige–Skoog (MS) solid medium at pH 5.8. The *OsD-LDH* RNAi and empty vector control (CK) transgenic lines were grown on the same medium supplemented with 30 mg L^-1^ hygromycin B (Roche, United States). For the MG treatment, plants were grown on the corresponding media supplemented with different concentrations of MG in the presence or absence of 2% sucrose.

For the illumination treatment, plants were grown on the corresponding media supplemented with 2% sucrose under a 14/10-h light/dark photoperiod (120 μmol photons m^-2^ s^-1^ or 1000 μmol photons m^-2^ s^-1^ for low and high light intensities, respectively). The light intensity was measured using a Quantum Light Meter (Spectrum, United States). All of the plants were grown in chambers at 28°C until evaluation. Statistical significance was assessed using a Student’s *t*-test.

For the NaCl treatment, plants were grown on the corresponding media for 2–3 weeks under a 14/10-h light/dark photoperiod (120 μmol photons m^-2^ s^-1^) at 27°C. Five leaf segments (about 5 cm) of each sample were cut and floated on deionized water or 200 mM NaCl for 3–5 days under the same conditions with continuous observation.

### MG, GSH, and D-Lactate Determination

Methylglyoxal content was estimated according to the method reported in a previous study (Mustafiz et al., 2010) with modifications. The absorbance was measured at 336 nm using a Tecan Infinite M200 PRO microplate reader.

Total and oxidized glutathione (GSSG) analyses were conducted according to a previous study (Mourad et al., 2000) with a GSH and GSSG Assay Kit (Beyotime, China) according to the manufacturer’s instructions. The total GSH content was measured based on the kinetic rate of OD_412_ using a Tecan Infinite M200 PRO microplate reader. The GSSG content was determined using the same method as the total GSH assay, after removal of reduced GSH with 2-vinylpyridine. The reduced GSH was calculated by subtracting the GSSG from the total GSH according to the following equation: total GSH - 2 × GSSG.

The D-lactate content was estimated by enzyme-linked immunosorbent assay (ELISA) using a plant D-lactate ELISA Kit (Fankebio, China) according to the manufacturer’s instructions with modifications. The absorbance was measured at 450 and 630 nm using a Tecan Infinite M200 PRO microplate reader. The D-lactate content was calculated according to the D-lactate standard curve after subtracting OD_630_ from OD_450_.

All of the assays were performed with at least three different extractions of the ground-up tissues and at least three determinations of each sample. Statistical significance was assessed using a Student’s *t*-test.

### Protein Extraction and Enzymatic Activity Assay

The enzymatic activities of glyoxalase I (Gly I) and II (Gly II) were determined as described previously (Mustafiz et al., 2010) with modifications. Young leaf tissues were ground in liquid nitrogen and 0.2 mL of protein extraction buffer (0.1 M K_3_PO_4_ buffer (pH 7.0), 50% glycerol, 16 mM MgSO_4_, 0.2 mM PMSF, and 0.1% Triton X-100) was added to 0.1 g of tissue sample. After vortexing and incubation on ice, the extract was centrifuged at 18,000 × *g* and 4°C for 15 min. The supernatant was assayed for protein content using the bicinchoninic acid (BCA) method and then immediately used for assessment of the Gly I and II activities. The absorbance was measured at 240 nm using a Tecan Infinite M200 PRO microplate reader.

To determine the aldo-keto reductase activity, protein extract was prepared as described previously (Turóczy et al., 2011) with minor modifications. The supernatant was assayed for protein content using the BCA method and then immediately used for assessment of the aldo-keto reductase activity. The absorbance was measured at 340 nm using a Tecan Infinite M200 PRO microplate reader. The reaction was initiated according to a previous method (Suekawa et al., 2016) by the addition of 20 μL of protein extract to a reaction mixture containing 50 mM Tris-HCl (pH 7.2), 0.1 mM NADPH, and 2 mM MG in a total volume of 200 μL. The absorbance was monitored for 2 min. The amount of oxidized NADPH was calculated using the molar extinction coefficient of NADPH (6.22 mM^-1^ cm^-1^).

All of the assays were performed with at least three different enzyme extractions of the ground-up tissues and at least three determinations of each sample. Statistical significance was assessed using a Student’s *t*-test.

### Estimation of Chlorophyll Content

All treated leaf segments were ground in liquid nitrogen and chlorophyll was extracted from these leaf powders with ice-cold 80% acetone, and the chlorophyll contents were determined as described previously (Lichtenthaler, 1987). Assays were performed with at least two different extractions of the ground-up tissues and at least three determinations of each sample. Statistical significance was assessed using a Student’s *t*-test.

## Results

### Identification and Cloning of Putative *OsD-LDH*

**FIGURE 1:**
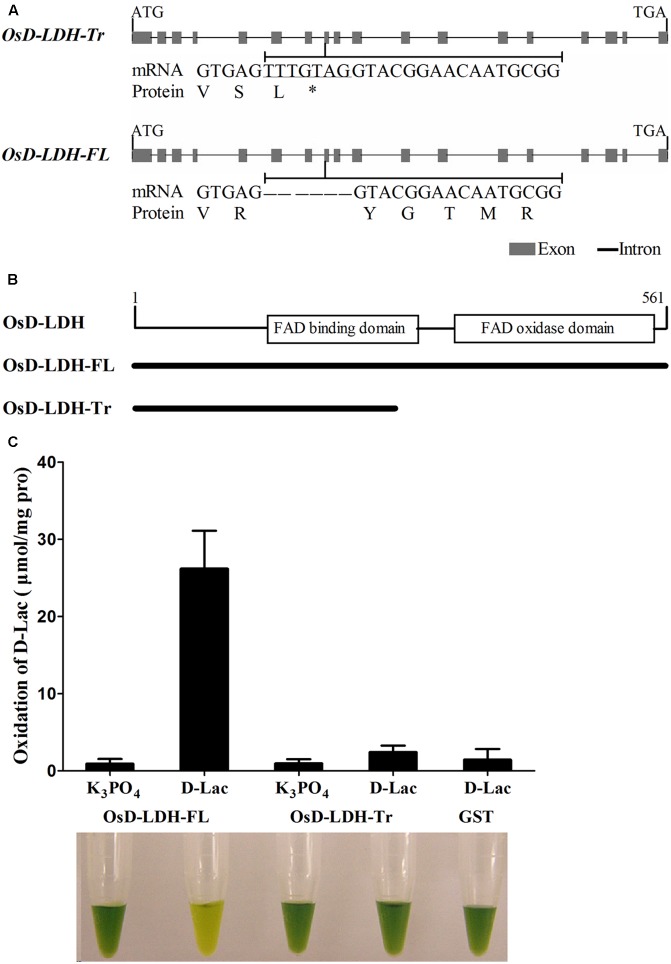
Gene structures, alternative splicing and enzymatic activities of *OsD-LDH.* **(A)** Gene structures of *OsD-LDH* and differences between two AS variants, *OsD-LDH-Tr* and *OsD-LDH-FL*. The 7-bp-sequence difference was indicated by underline. *OsD-LDH-Tr*, the truncated *OsD-LDH* variant; *OsD-LDH-FL*, the full-length *OsD-LDH* variant; ^∗^, stop codon. **(B)** Schematic diagram of FAD-linked domains in OsD-LDH. The protein lengths OsD-LDH-Tr and OsD-LDH-FL were shown below. **(C)** Comparison of catalytic properties between OsD-LDH-Tr and OsD-LDH-FL. The purified OsD-LDH-Tr and OsD-LDH-FL proteins were added with substrate (D-Lac) or without substrate (K_3_PO_4_) in the reaction mixtures, respectively, to examine their activities. The purified GST protein was used as control. The reactions were performed at 25°C for 15 min using PMS-DCIP as the electron acceptor, and then the reaction mixtures were examined at OD_600_. The reaction mixtures were photographed after the reactions finished. From left to right, they were combination of (OsD-LDH-FL+K_3_PO_4_), (OsD-LDH-FL+D-Lac), (OsD-LDH-Tr+K_3_PO_4_), (OsD-LDH-Tr+D-Lac), and (GST+D-Lac), respectively. Values represent the mean ± SD (*n* = 6). D-Lac, D-lactate; GST, Purified Glutathione S-transferase as negative control.

To identify candidate D-lactate dehydrogenase or GDH genes, the rice genome was searched using the sequences of *Saccharomyces cerevisiae* D-lactate dehydrogenase (ScDLD1) (Lodi and Ferrero, 1993) and *A. thaliana* D-lactate/GDH (AtD-LDH or AtGDH) (Bari et al., 2004; Engqvist et al., 2009) and the NCBI TBLASTN tool. The top hit in both cases was LOC_Os07g06890, which is located on the short arm of chromosome 7 and encodes a protein belonging to the FAD-linked oxidase family. LOC_Os07g06890 was found to exhibit 18.2 and 69.8% identity and 25.3 and 79.5% similarity to ScDLD1 and AtD-LDH/AtGDH, respectively. This high level of identity and similarity indicated that LOC_Os07g06890 was a putative D-LDH/GDH in rice. However, the predicted amino acid sequence was interrupted by a premature termination codon (PTC), resulting in a peptide of only 254 amino acids in length, according to both the RefSeq RNA database in GenBank (NM_001065487) and the KOME database (AK067700). However, it was believed that the rice genome retained the ability to encode D-LDH/GDH. As such, the putative *OsD-LDH/GDH* was amplified from *O. sativa* L. ssp. *indica* ‘93-11’ and the PCR products were sequenced. Fortunately, another transcript was obtained besides the transcript AK067700, indicating the occurrence of alternative splicing (AS). The AK067700 transcript and the newly found transcript were referred to as the types I and II variants, respectively (Supplementary Table S1). A 7-bp sequence (TTTG*TAG*) deletion was detected in exon 8 of the type II variant due to an alternative donor splice site, which resulted in a full-length protein, while the type I variant retained the 7-bp sequence (TTTG*TAG*) and formed a PTC (shown in italics), resulting in the truncated protein (**Figures 1A,B** and Supplementary Figure S1). The corresponding proteins are referred to as truncated OsD-LDH (OsD-LDH-Tr) and full-length OsD-LDH (abbreviated OsD-LDH-FL), respectively. *OsD-LDH-Tr* encodes a peptide of 254 amino acids, while *OsD-LDH-FL* encodes a peptide of 561 amino acids. The amino acid sequence of OsD-LDH-Tr is identical to that of OsD-LDH-FL except for the last two amino acids (Supplementary Figure S1). Both sequences were aligned with the known D-LDH/GDH and the full-length protein showed the highest similarity with AtD-LDH/AtGDH (Supplementary Figure S1). Unexpectedly, when the type I sequence was used to search against the expressed sequence tag database in NCBI, these two types of AS were also found to be present in *Aquilegia*, *Carica papaya*, and *Zea mays*, revealing that the 7-bp sequence (TTTG*TAG*) differences of AS were conserved both in dicots and monocots (Supplementary Table S2). Phylogenetic analysis revealed that mammals, nematodes, algae, fungi, and bacteria also possess orthologous sequences, in addition to higher plants (Supplementary Figure S2 and Supplementary Table S3) (Cristescu and Egbosimba, 2009), which were categorized into different clades.

### Catalytic Properties of OsD-LDH

The OsD-LDH-FL protein was predicted to possess an FAD-binding domain (IPR004113) between amino acids 140 and 277 and an FAD-oxidase domain (IPR006094) between amino acids 313 and 554 based on SWISS-MODEL (Arnold et al., 2005) (**Figure 1B** and Supplementary Figure S1). Although OsD-LDH-Tr retained the same amino acids as the first 252 amino acids of OsD-LDH-FL (**Figures 1A,B** and Supplementary Figure S1) according to the prediction, OsD-LDH-Tr lacked the entire C-terminal FAD-oxidase domain and part of the FAD-binding domain (**Figure 1B** and Supplementary Figure S1).

To investigate the catalytic properties of OsD-LDH, OsD-LDH-FL and OsD-LDH-Tr were expressed in *E. coli*. The recombinant proteins, GST-OsD-LDH-FL (87 kDa) and GST-OsD-LDH-Tr (55 kDa), were detected in both the supernatant and pellets of the induced cells (data not shown). After the recombinant proteins had been purified from the supernatants and confirmed by western blot analysis (Supplementary Figure S3), the catalytic properties were determined immediately. The recombinant OsD-LDH-FL protein rapidly catalyzed the oxidation of D-lactate to pyruvate in the presence of phenazine methosulfate and 2,6-dichlorophenolindophenol (PMS-DCIP) as an electron acceptor (**Figure 1C**). However, the recombinant OsD-LDH-Tr failed to oxidize D-lactate as the negative control (GST), which indicated that OsD-LDH-Tr had lost most of its catalytic capability due to the absence of the C-terminal FAD-oxidase domain (**Figure 1C**). These results suggested that full-length OsD-LDH was able to function as a D-lactate dehydrogenase.

**FIGURE 2:**
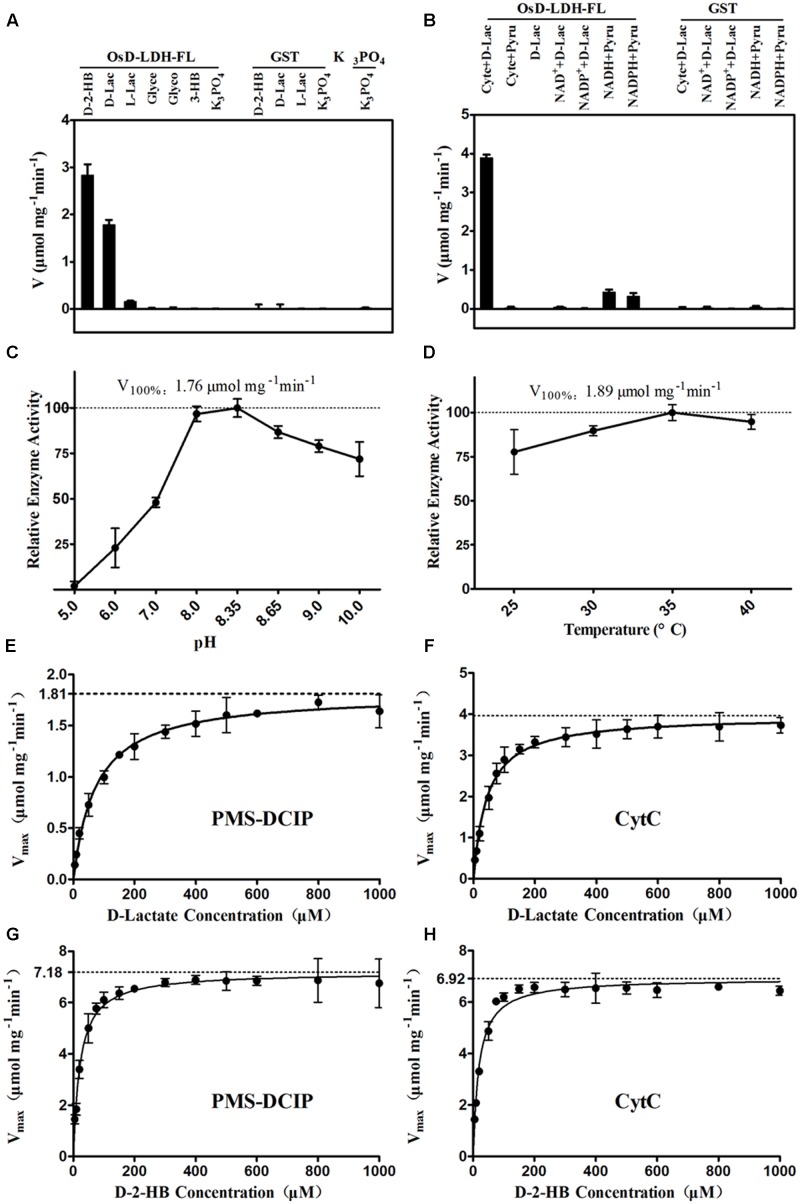
Catalytic properties of the recombinant OsD-LDH-FL. **(A)** Substrate specificity screening of OsD-LDH, performed at 25°C and pH 8.35 using Cyt c as the electron acceptor. **(B)** Electron acceptors screening of OsD-LDH, performed at 25°C and pH 8.35. **(C)** pH optimum of OsD-LDH, performed at 25°C using PMS-DCIP as the electron acceptor. **(D)** Temperature optimum of OsD-LDH, performed at pH 8.35 using PMS-DCIP as the electron acceptor. **(E–H)** Kinetic parameters of OsD-LDH toward D-lactate and D-2-HB using PMS-DCIP and Cyt c as electron acceptors, respectively; *V*_max_ and *K*_M_ were determined using a Hanes–Woolf plot method and all experiments were performed at 30°C and pH 8.35. D-2-HB, D-2-hydroxybutyrate; D-Lac, D-lactate; L-Lac, L-lactate; Glyce, Glycerol; Glyco, Glycolate; 3-HB, 3-hydroxybutyrate; GST, Purified Glutathione *S*-transferase as negative control. Values represent the mean ± SD (*n* = 3).

The catalytic properties of the full-length OsD-LDH were then investigated in more detail. The substrate specificity screening using several related substances revealed that OsD-LDH exhibited very high catalytic efficiencies for D-2-hydroxybutyrate (D-2-HB) and D-lactate, low catalytic efficiency for L-lactate, and no activities for glycerol, glycolate, or 3-hydroxybutyrate. The catalytic efficiencies of OsD-LDH for D-2-HB and L-lactate were approximately 150 and 10% of that for D-lactate, respectively (**Figure 2A**). In the screening experiments for potential electron acceptors, Cyt *c* functioned as an electron acceptor for the oxidation of D-lactate, whereas NAD^+^ and NADP^+^ did not. However, OsD-LDH catalyzed the reduction of pyruvate to lactate in the presence of NADH or NADPH, albeit with very low efficiency (**Figure 2B**). The optimal pH and temperature were determined to evaluate the kinetic properties of OsD-LDH, and the enzyme was found to be active over relatively broad ranges of pH and temperature centered at pH 8.35 and 35°C, respectively (**Figures 2C,D**).

To evaluate the affinity and catalytic efficiency of OsD-LDH, the kinetic parameters of the recombinant full-length OsD-LDH were determined for its two main substrates, D-lactate and textscd-2-HB, using either PMS-DCIP or Cyt *c* as the electron acceptor (**Figures 2E–H**). When PMS-DCIP was used as the electron acceptor, the affinity and catalytic efficiency of OsD-LDH for D-lactate and D-2-HB were very high, with *K*_M_ values of 68.19 and 14.87 μM and *V*_max_ values of 1.79 and 6.92 μmol mg^-1^ min^-1^, respectively. From these data, the *k*_cat_ values were calculated to be 2.60 and 10.03 s^-1^, respectively, affording *k*_cat_/*K*_M_ values of 3.81 × 10^4^ M^-1^ s^-1^ and 6.75 × 10^5^ M^-1^ s^-1^, respectively (**Figures 2E,G**, **Table 1**, and Supplementary Figures S8A,C). When Cyt *c* was used as the electron acceptor, the affinity and catalytic efficiency of OsD-LDH for D-2-HB were similar to those obtained using PMS-DCIP. However, the affinity and catalytic efficiency of OsD-LDH for D-lactate almost doubled compared with those obtained using PMS-DCIP. The *K*_M_ value decreased to 41.4 μM and the *V*_max_ value increased to 3.98 μmol mg^-1^ min^-1^. From these data, the *k*_cat_ was calculated to be 5.77 s^-1^, giving a *k*_cat_/*K*_M_ value of 1.39 × 10^5^ M^-1^ s^-1^ (**Figures 2F,H**, **Table 1**, and Supplementary Figures S8B,D), which is probably because Cyt *c* is the electron acceptor *in vivo*. In summary, these results demonstrated that full-length OsD-LDH acted as a highly efficient Cyt-*c*-dependent D-lactate dehydrogenase.

**Table 1:** Kinetic parameters of the recombinant full-length OsD-LDH.

	D-LACTATE			D-2-HB		
		
	*K*_M_ (μM)	*k*_cat_ (S^-1^)	*k*_cat_*/K*_M_ (M^-1^S^-1^)	*K*_M_ (μM)	*k*_cat_ (S^-1^)	*k*_cat_*/K*_M_ (M^-1^S^-1^)
With PMS-DCIP	68.19 ± 5.38	2.60 ± 0.06	3.81 × 10^4^	14.87 ± 0.87	10.03 ± 0.23	6.75 × 10^5^
With Cyt c	41.40 ± 2.93	5.77 ± 0.09	1.39 × 10^5^	11.02 ± 0.34	9.58 ± 0.09	8.69 × 10^5^


### *OsD-LDH* Shows Preferential Transcript Accumulation in Flag Leaves

**FIGURE 3:**
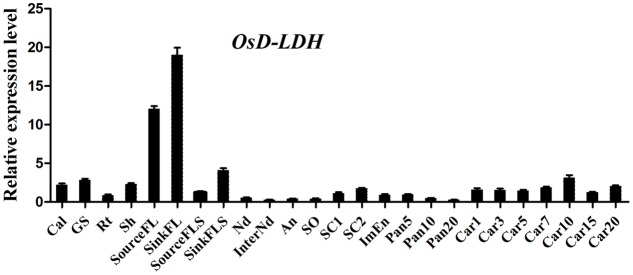
Expression profile of *OsD-LDH* gene at different developmental stages. Cal, callus; GS, 7–day-germinating seeds; Rt, 14-day-old roots; Sh, 14-day-old shoots; sourceFL, source flag leaves, fully expanded flag leaves harvested from plants 1 week after heading; SinkFL, sink flag leaves, unexpanded flag leaves harvested approximately 3 weeks before heading; SinkFLS, sink flag leaf sheaths, flag leaf sheaths harvested from plants 1 week before heading; SourceFLS, source flag leaf sheaths, flag leaf sheaths harvested from plants 1 week after heading; Nd, nodes; InterNd, internodes; An, anther; SO, stigma and ovary; SC1, 3 days seeds coat after heading; SC2, 15 days seeds coat after flowering; ImEn, 15 days immature endosperm after flowering; Pan5/10/20, panicles harvested before heading at lengths of 5/10/20 cm; Car1/3/5/7/10/15/20, 1/3/5/7/10/15/20 days caryopses after flowering. Values represent the mean ± SD (*n* = 3).

Following its identification as a Cyt-*c*-dependent D-lactate dehydrogenase, the accumulation of *OsD-LDH* transcripts in various rice tissues was measured by qRT-PCR to evaluate its physiological functions in plants. Primers were designed at the 3′ end of *OsD-LDH* cDNA to detect the total transcripts. The results showed that *OsD-LDH* was expressed in all of the examined tissues at different developmental stages from germination to grain filling (**Figure 3**). The *OsD-LDH* transcripts accumulated to the highest levels in the flag leaves, while moderate levels were detected in the callus, germinating seeds, shoots, flag leaf sheaths, seed coats, and caryopses, and very low levels were found in young roots, stems, anthers, pistils, immature endosperms, and panicles before heading. These results suggested that the prominent functions of *OsD-LDH* occurred in the leaves. The *OsD-LDH* transcript levels increased during the early stage of grain filling (5–10 days after flowering) and then declined, suggesting a probable role in early grain filling.

### The N-Terminal Domain of OsD-LDH Targets It to the Mitochondrion

**FIGURE 4:**
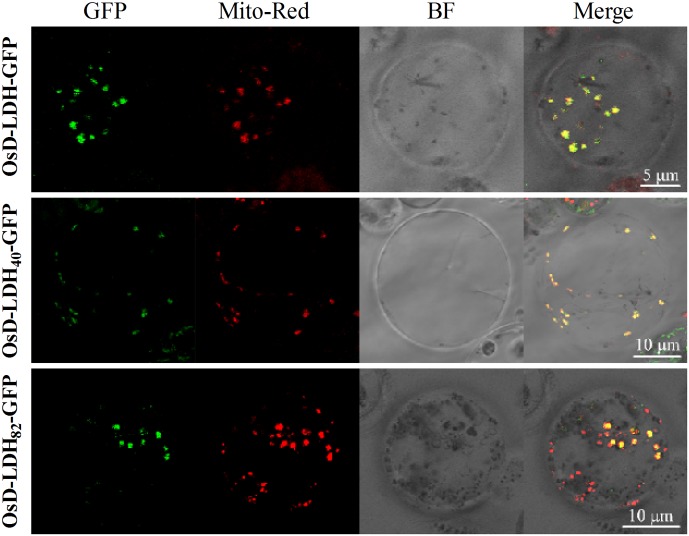
Subcellular localization of OsD-LDH in protoplasts of transgenic rice plants. OsD-LDH-GFP, OsD-LDH_40_-GFP, and OsD-LDH_82_-GFP, GFP fused to full-length OsD-LDH, first N-terminal 40 and 82 amino acids of OsD-LDH, respectively. All the signal peptides were fused in-frame to the N-terminus of GFPs. Mito-Red, mitochondrion specific dye Mito-Tracker Red; BF, Bright field. The bars were indicated in the figures.

The subcellular localization of OsD-LDH was analyzed using the full-length OsD-LDH-GFP fusion protein from transgenic T1 rice plants to investigate its potential roles *in vivo*. Protoplasts were prepared from transgenic seedlings and mitochondria were specifically stained with MitoTracker Red. The confocal laser images revealed colocalization of the OsD-LDH-GFP fusion protein (**Figure 4**, in green) and stained mitochondria (in red), which indicated that OsD-LDH was located in the mitochondria.

To determine the location and length of the signal peptide, the full-length OsD-LDH sequence was analyzed using the SignalP algorithm (version 4.1) (Emanuelsson et al., 2007). The prediction indicated the existence of two potential signal peptides with 11 and 15 N-terminal amino acids. The N-terminal 20, 40, and 82 amino acids and the C terminus of full-length OsD-LDH (without the first 79 N-terminal amino acids) were fused with GFP to generate the fusion proteins OsD-LDH_20_-GFP, OsD-LDH_40_-GFP, OsD-LDH_82_-GFP, and OsD-LDH_C′_-GFP, respectively. The confocal laser images showed that the OsD-LDH_40_-GFP and OsD-LDH_82_-GFP fusion proteins colocalized with MitoTracker Red (**Figure 4**), which indicated that the first 40 amino acids of OsD-LDH targeted the protein to the mitochondria. The green fluorescence of the OsD-LDH_C′_-GFP fusion protein was distributed throughout the cytoplasm similar to the GFP control (Supplementary Figure S4), further confirming that the signal peptide of OsD-LDH was located at the N terminus. However, the green fluorescence of the OsD-LDH_20_-GFP fusion protein was also distributed throughout the cytoplasm similar to the GFP control (Supplementary Figure S4). This result was inconsistent with the SignalP prediction, indicating that the first 20 amino acids of OsD-LDH alone failed to target the protein to the mitochondria. Overall, as a result of the N-terminal signal peptide in the first 40 amino acids, OsD-LDH localized to the mitochondria.

### *OsD-LDH* RNAi Plants Are Sensitive to Treatment with MG

To functionally analyze the roles of *OsD-LDH* in rice, the *OsD-LDH* RNAi construct was generated against the 493-bp fragment of the *OsD-LDH* cDNA. Thirty-three independent lines of hygromycin-resistant T0 transgenic rice seedlings were obtained, transferred to soil, and grown in the field to obtain T1 progeny for the functional analysis. These *OsD-LDH* RNAi transgenic lines exhibited no obvious differences during the vegetative or reproductive stages compared with the wild type (WT) under field conditions (data not shown). The qRT-PCR analysis revealed a considerable decline of *OsD-LDH* transcripts in young leaves in 13 out of 20 independent lines compared with the WT. Among these 13 lines, the expression of *OsD-LDH* was strongly suppressed in lines 2, 3, 9, 29, and 31 (Supplementary Figure S5A), which were used for further analyses.

**FIGURE 5:**
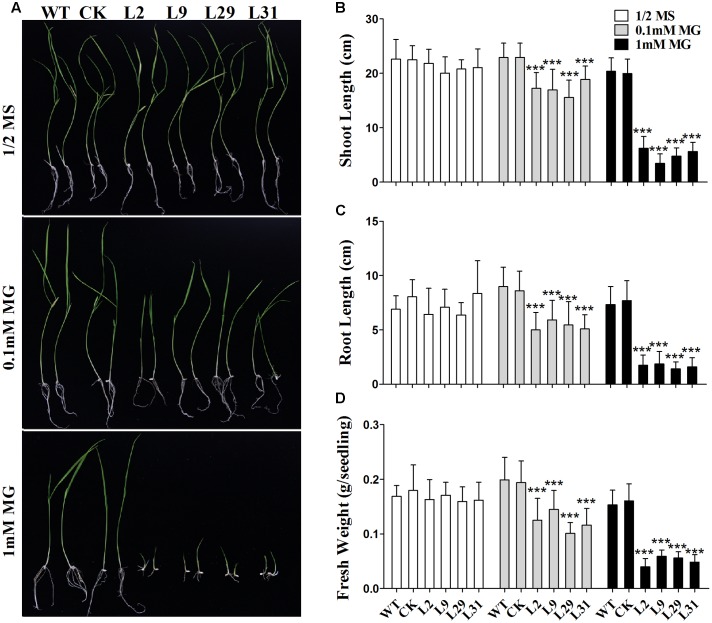
Effect of MG on 2-week-old seedlings. **(A)** Two-week-old seedlings were grown on ½ MS solid media containing 2% sucrose for wild type plants or selected with 30 mg L^-1^ hygromycin B for transgenic plants; MG treatments were in the same media supplemented with 0.1 mM or 1 mM MG, respectively. **(B–D)** Determination of shoot length, root length, and fresh weight of the WT, CK, and *OsD-LDH* RNAi transgenic lines. Values represent the mean ± SD (*n* = 30) (^∗∗∗^*P* < 0.001). WT, wild type plants; CK, empty vector control; L2, L9, L29, and L31, *OsD-LDH* RNAi plants.

Because OsD-LDH is responsible for converting D-lactate to pyruvate and D-lactate is mainly derived from MG, to further investigate the function of OsD-LDH in MG metabolism, the WT, empty vector control (CK), and *OsD-LDH* RNAi plants were germinated on ½ MS media supplemented with 0.1 or 1 mM MG. There were no obvious differences between 1-week-old or 2-week-old WT and *OsD-LDH* RNAi plants on ½ MS media (Supplementary Figure S5B and **Figure 5A**), similar to that observed under field conditions. However, the *OsD-LDH* RNAi seedlings displayed significant growth inhibition on medium supplemented with 0.1 mM MG (Supplementary Figure S5B and **Figure 5A**). The growth inhibition of 1-week-old *OsD-LDH* RNAi seedlings was more severe when they were grown on medium supplemented with 1 mM MG, and no emergence of radicles was observed (Supplementary Figure S5B). Statistical analyses of 2-week-old plants grown on media supplemented with 1 mM MG revealed that the shoot and root lengths were much shorter in lines 2, 9, 29, and 31 compared with the WT and CK plants (**Figures 5B,C**). In addition, the fresh weight per plant declined greatly (**Figure 5D**). To eliminate the influence of the carbon source on the growth of *OsD-LDH* RNAi plants, we repeated this experiment under the same conditions except that the media were not supplemented with sucrose. The phenotypes of *OsD-LDH* RNAi seedlings under MG treatment were similar whether or not they were supplemented with sucrose (Supplementary Figure S6). These results suggested that the silencing of OsD-LDH led to severe growth inhibition under MG treatment, which indicated that OsD-LDH is involved in MG metabolism.

### MG Stress Disrupts MG, GSH, and D-Lactate Homeostasis and Affects the Glyoxalase System and Aldo-Keto Reductase Activity in *OsD-LDH* RNAi Plants

**FIGURE 6:**
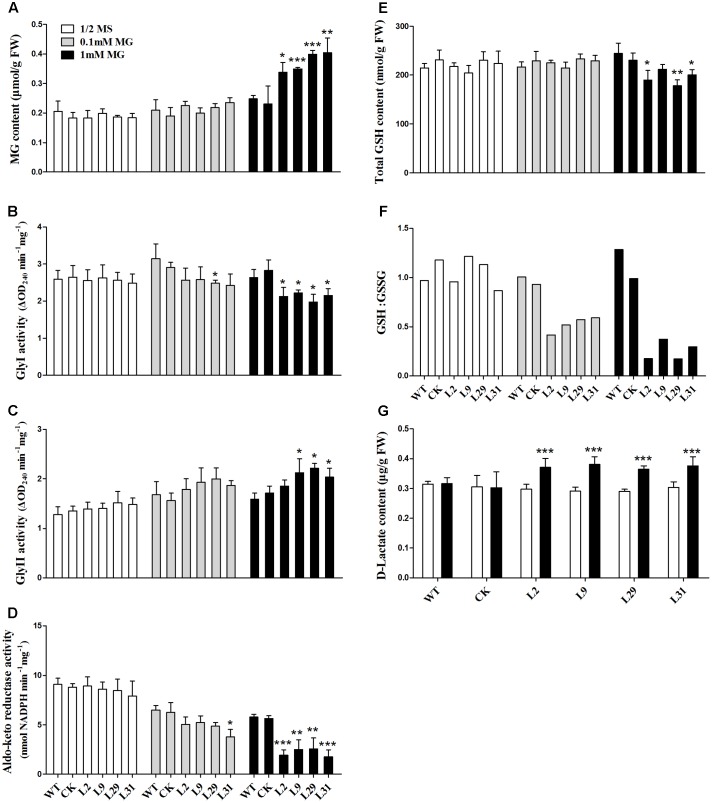
Effect of MG stress on the levels of MG, GSH, D-lactate, glyoxalase system and aldo-keto reductases *in vivo*. **(A)** MG contents in young leaves under ½ MS media or supplemented with 0.1 mM or 1 mM MG. **(B,C)** Activities of Gly I and Gly II in the same sample of **A**, respectively. **(D)** Activity of aldo-keto reductases (OsAKRs) in the same sample of **A**. **(E,F)** Total GSH contents and reduced GSH:GSSG ratio in the same sample of **(A)**, respectively. **(G)** D-lactate contents in the same sample of **(A)**. Values represent the mean ± SD (*n* = 30) (^∗^*P* < 0.05, ^∗∗^*P* < 0.01, ^∗∗∗^*P* < 0.001). WT, wild type plants; CK, empty vector control; L2, L9, L29, and L31, *OsD-LDH* RNAi plants; Gly I, glyoxalase I; Gly II, glyoxalase II.

To investigate the biochemical basis of the growth inhibition observed in *OsD-LDH* RNAi seedlings treated with MG, the MG content was measured in 2-week-old plants. The MG levels were similar in the WT, CK, and *OsD-LDH* RNAi seedlings on ½ MS media (**Figure 6A**) and were slightly increased in response to 0.1 mM MG treatment (**Figure 6A**). However, treatment with 1 mM MG significantly increased the levels of MG in the *OsD-LDH* RNAi seedlings (**Figure 6A**). Because MG in plants is degraded mainly by the glyoxalase system (Yadav et al., 2005), the enzymatic activities of glyoxalases I and II were measured in the young leaves of 2-week-old plants. For glyoxalase I, there were no obvious differences between *OsD-LDH* RNAi plants and the WT or CK plants grown on ½ MS media, whereas the activity diminished and decreased significantly in *OsD-LDH* RNAi plants exposed to 0.1 and 1 mM MG, respectively (**Figure 6B**). The decreased activity of glyoxalase I was consistent with the increased level of MG in *OsD-LDH* RNAi plants. For glyoxalase II, the activity increased slightly in *OsD-LDH* RNAi plants compared with the WT or CK plants grown on ½ MS media or media supplemented with 0.1 mM MG and increased significantly under treatment with 1 mM MG (**Figure 6C**). The activity of glyoxalase II showed a profile that was opposite to that of glyoxalase I. Another MG detoxification pathway involves aldo-keto reductases, and therefore the activity of aldo-keto reductases was measured under the same treatment conditions. Surprisingly, the aldo-keto reductase activity was also inhibited in the *OsD-LDH* RNAi plants under MG treatment (**Figure 6D**). These results indicated that both the activity of glyoxalase system and the aldo-keto reductases were affected in the *OsD-LDH* RNAi plants under MG treatment, both of which led to the accumulation of MG.

Because GSH is involved in MG metabolism, it was important to analyze whether the GSH content was affected in the *OsD-LDH* RNAi plants. The levels of total GSH were similar in the 2-week-old WT, CK, and *OsD-LDH* RNAi plants regardless of whether they were grown on ½ MS media or media supplemented with 0.1 mM MG (**Figure 6E**). In response to the 1 mM MG treatment, the levels of total GSH decreased significantly in the *OsD-LDH* RNAi transgenic lines 2, 29, and 31 compared to the WT plants (**Figure 6E**). Further analysis of the GSH:GSSG ratio showed that there were no obvious differences between the *OsD-LDH* RNAi and the WT or CK plants on ½ MS media, whereas upon exposure to 0.1 mM MG the GSH:GSSG ratio decreased dramatically in the *OsD-LDH* RNAi plants to half of the level detected in the WT or CK plants, and an even greater decrease was observed upon exposure to 1 mM MG (**Figure 6F**). These results indicated that GSH homeostasis was disrupted in *OsD-LDH* RNAi plants, based on the large decrease in reduced glutathione.

Finally, the D-lactate content was analyzed to determine whether the levels of this compound were affected in *OsD-LDH* RNAi plants under MG stress. The D-lactate levels were found to be similar in the WT, CK, and *OsD-LDH* RNAi seedlings on ½ MS media (**Figure 6G**). However, treatment with 1 mM MG significantly increased the D-lactate levels in *OsD-LDH* RNAi seedlings (**Figure 6G**). These results indicated that under MG stress D-lactate accumulated *in vivo* when *OsD-LDH* was silenced.

### *OsD-LDH* RNAi Plants Are Not Sensitive to Strong Light

**FIGURE 7:**
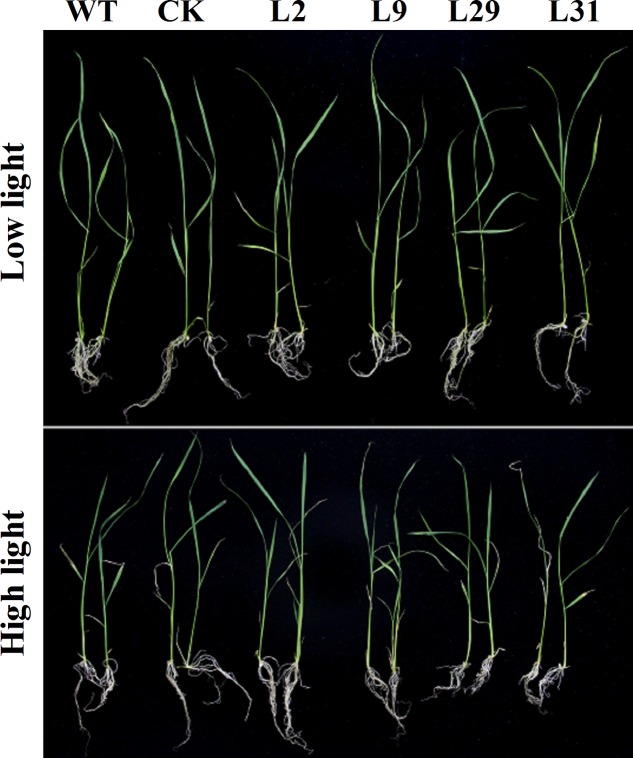
Effect of 2-week-old seedlings grown under different light intensity. Two-week-old seedlings were grown on ½ MS solid media containing 2% sucrose for wild type plants or selected with 30 mg L^-1^ hygromycin B for transgenic plants. They were placed under different light intensity. Low light, light intensity was 120 μmol photons m^-2^ s^-1^; High light, light intensity was 1000 μmol photons m^-2^ s^-1^. WT, wild type plants; CK, empty vector control; L2, L9, L29, and L31, *OsD-LDH* RNAi plants.

To investigate whether OsD-LDH participates in photorespiration, we studied *OsD-LDH* RNAi seedlings grown under different light intensities. Because strong light will lead to photorespiration in plants (Gerbaud and André, 1980), we conducted experiments at a light intensity of 1000 μmol photons m^-2^ s^-1^ to represent photorespiration conditions and at a light intensity of 120 μmol photons m^-2^ s^-1^ as the control. The seedlings grown under strong light showed greener leaves but wilted leaf tips compared with the seedlings grown under low light (**Figure 7**). The shoot lengths of 2-week-old seedlings grown under strong light were slightly shorter than those grown under low light and the root lengths were slightly longer, although the fresh weights were similar (Supplementary Figures S7A–C). However, there were no significant differences between the *OsD-LDH* RNAi seedlings and the WT or CK grown under strong light (**Figure 7** and Supplementary Figure S7), which indicated that silencing *OsD-LDH* did not affect the growth of seedlings under photorespiration conditions.

### *OsD-LDH* RNAi Plants Are Sensitive to Salt Stress

**FIGURE 8:**
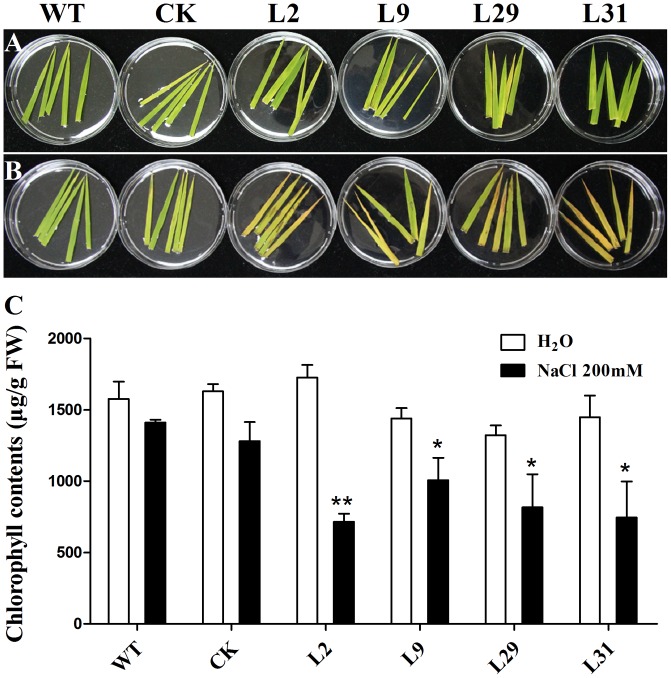
Effect of salt stress on the detached leaves. **(A,B)** The 5-cm-length leaves were cut from about 3 weeks old seedlings and floated on deionized water and 200 mM NaCl for 5 days, respectively. **(C)** Chlorophyll contents in the leaves of **(A,B)**. Values represent the mean ± SD (*n* = 10) (^∗^*P* < 0.05, ^∗∗^*P* < 0.01). WT, wild type plants; CK, empty vector control; L2, L9, L29, and L31, *OsD-LDH* RNAi plants; FW, fresh weight.

To investigate whether OsD-LDH participates in abiotic stresses because of its influence on MG degradation, we tested the response of *OsD-LDH* RNAi plants to salt stress using the detached leaf senescence assay. After being floated on 200 mM NaCl for 5 days, the leaf segments of *OsD-LDH* RNAi plants showed an early senescence compared to the WT or CK, but the leaf segments being floated on deionized water exhibited no obvious differences between them (**Figures 8A,B**). Investigations of chlorophyll contents indicated that under NaCl treatment, chlorophyll contents decreased drastically in *OsD-LDH* RNAi leaf segments compared to the WT or CK, while under deionized water treatment, the chlorophyll contents of *OsD-LDH* RNAi leaf segments retained the similar levels as the WT or CK (**Figure 8C**). These results indicated that silencing of OsD-LDH led to the detached leaf segments being sensitive to salt stress.

## Discussion

In this study, we have described the isolation and characterization of a highly efficient mitochondrial Cyt-*c*-dependent D-lactate dehydrogenase in rice, OsD-LDH, which is an FAD-linked protein that is evolutionarily conserved in prokaryotes and eukaryotes. Alternative splicing events were detected in the transcripts of *OsD-LDH* and the corresponding proteins possessed distinct enzymatic activities. Functional analysis revealed that *OsD-LDH* was responsible for the oxidation of D-lactate produced by MG metabolism.

As a result of its ability to generate multiple transcripts and potentially different proteins from the same gene, AS is considered as a key post-transcriptional regulatory mechanism for expanding the diversity of proteins in higher eukaryotes (Carvalho et al., 2013). It remains unclear why the truncated OsD-LDH translated from one of the AS variants has no or extremely low D-lactate dehydrogenase activity (**Figure 2C**). However, the conservation of the two types of AS, type I and type II, in rice, *Aquilegia*, *C. papaya* and *Z. mays* (Supplementary Table S2) indicates that these two types of AS events might be involved in some ancient and important biological processes. Possible explanations for this are that the truncated OsD-LDH might participate in some other biological reactions or represent one way of expanding protein diversity, although these possibilities need further investigation.

The enzymatic properties of the full-length OsD-LDH suggest that it acts as a Cyt-*c*-dependent D-lactate dehydrogenase in rice, displaying a higher affinity and catalytic efficiency than most of the known Cyt-*c*-dependent D-LDHs (**Figure 2** and **Table 1**) (Ogata et al., 1981; Horikiri et al., 2004; Atlante et al., 2005; Engqvist et al., 2009). *OsD-LDH* RNAi transgenic plants showed severe growth inhibition under MG treatment (Supplementary Figure S5B and **Figure 5A**), which was mostly caused by the increased levels of MG and D-lactate and the decreased level of reduced GSH (**Figures 6A,E–G**). The changes in these metabolite contents arose from silencing of OsD-LDH, which disturbed the function of the glyoxalase and aldo-keto reductase systems under MG treatment (**Figures 6B,C**). These phenotypes were similar to the sensitivities of *Gly I*-antisense tobacco plants grown under stress conditions (Reddy and Sopory, 1999; Yadav et al., 2005) and to the inability of *S. cerevisiae* strains deficient in Gly I or Gly II to grow on media supplemented with high concentrations of MG (Inoue and Kimura, 1996; Bito et al., 1997). These results demonstrated that the D-lactate dehydrogenase activity of OsD-LDH *in vitro* was consistent with its function in MG metabolism *in vivo*. Furthermore, there are 11 putative *Gly I* and 3 putative *Gly II* genes in the rice genome, of which *OsGLY I*-2, *OsGLY I-*7, *OsGLY I-*9, and *OsGLY II-*3 are ubiquitously expressed with higher levels in leaves (Mustafiz et al., 2011). A similar expression pattern was observed for *OsD-LDH* (**Figure 3**), indicating that OsD-LDH functioned in a coordinated manner with the glyoxalase system. Furthermore, prediction of the subcellular localization of the glyoxalase system in rice using the TargetP algorithm (Emanuelsson et al., 2007) showed that OsGly I-1, 2, 6.4, and 7, as well as OsGly II-1, were probably targeted to the mitochondria (Supplementary Table S4). The overlapping localization of OsD-LDH and the glyoxalase system permitted the participation of OsD-LDH in the MG degradation pathway. Recently, OsGLYI-11.2 was characterized as an efficient enzyme that converts MG to SLG in rice. Its transcripts were induced by MG exposure (Mustafiz et al., 2014), which was consistent with our results showing that the Gly I activities of the WT and CK increased under MG treatment. However, in the *OsD-LDH* knockdown lines, the Gly I activity was inhibited under MG treatment, indicating that silencing of OsD-LDH changed the Gly I behavior. Although OsGLYII-2 was localized to the cytoplasm, its activity was inhibited by D-lactate and GSH *in vitro*, demonstrating end-product feedback mechanisms (Ghosh et al., 2014). However, our results showed that increasing D-lactate levels caused by silencing of *OsD-LDH* did not inhibit the Gly II activities. This might be because the D-lactate levels (micromolar level) *in vivo* (Ohmori et al., 1997) are far less than those in the experiments (millimolar level) *in vitro* (Ghosh et al., 2014). It is still unclear why Gly I and II displayed reverse trends of activities in the *OsD-LDH* knockdown lines under MG treatment. Interactions between OsD-LDH and Gly I/II are one of the possible reasons. Another surprising result was the severe inhibition of aldo-keto reductase activity in *OsD-LDH* knockdown lines under MG treatment. Acting as multi-tasking soldiers involved in a diverse range of plant metabolic processes and stress defenses, plant aldo-keto reductases can reduce a broad spectrum of substrates ranging from simple sugars to potentially toxic aldehydes, as described in a recent review (Sengupta et al., 2015). MG is one of the substrates for aldo-keto reductases and MG treatment would cause loss of the aldo-keto reductase activity (Saito et al., 2013), which was consistent with our results. As the concentration of MG increased, the aldo-keto reductase activity decreased (**Figure 6D**). However, it remains unclear why the activity of the aldo-keto reductases was severely inhibited in the *OsD-LDH* knockdown lines under MG treatment compared with the WT or CK (**Figure 6D**). The influence of OsD-LDH on the aldo-keto reductases may be attributable to interactions between OsD-LDH and aldo-keto reductases or some kind of feedback mechanism. Further work needs to be performed to clarify this. Besides, silencing of OsD-LDH caused the sensitivity to NaCl treatment (**Figure 8**), which demonstrated that OsD-LDH was involved in response to salt stress. These results further support the involvement of OsD-LDH in MG metabolism Because OsD-LDH plays a similar role as the glyoxalase system in response to salt stress (Bhomkar et al., 2008; Singla-Pareek et al., 2008; Mustafiz et al., 2011; Álvarez Viveros et al., 2013). Overall, these findings support the link of OsD-LDH to the glyoxalase system and aldo-keto reductases in rice.

Glycolate is a product of photorespiration caused by excessive light exposure or low CO_2_ concentration (Gerbaud and André, 1980). OsD-LDH exhibited no dehydrogenase activity for glycolate *in vitro* (**Figure 2A**). This was consistent with the observation that the *OsD-LDH* RNAi seedlings showed no differences compared with the WT or CK under intense light *in vivo* (**Figure 7**). These results suggested that OsD-LDH did not behave as a GDH involved in photorespiration in rice, which is in contrast to its homologous enzyme in *Arabidopsis*. In *Arabidopsis*, the enzyme showed both GDH and D-lactate dehydrogenase activities (AtGDH/AtD-LDH) (Bari et al., 2004; Engqvist et al., 2009). Moreover, the GDH activity of AtGDH/AtD-LDH contributed to photorespiration (Niessen et al., 2007, 2012) and the D-lactate dehydrogenase activity of AtGDH/AtD-LDH contributed to the glyoxalase system (Engqvist et al., 2009). The difference between OsD-LDH and AtGDH/AtD-LDH might be because of the divergence of monocotyledons and dicotyledons. Very recently, AtD-LDH was confirmed to use D-lactate but not glycolate as a substrate *in vivo* in *Arabidopsis* mitochondria (Maurino et al., 2016). Regardless of the discrepancy in *Arabidopsis*, OsD-LDH was involved in the glyoxalase system but not in photorespiration, which supports the point of view that plants do not possess a GDH (Maurino and Engqvist, 2015).

In summary, we have identified a Cyt-*c*-dependent D-lactate dehydrogenase in rice that contributes to MG metabolism and salt stress but not to photorespiration. Exploration of its responses to other environment stressors would help to elucidate the function of this D-lactate dehydrogenase in plants.

## Author Contributions

BA and YL conceived and designed the experiments. BA, JL, XD, SC, CO, and HS performed the experiments. BA analyzed the data and wrote the manuscript. BA, JY, and YL revised the manuscript. JL, XD, JY, and YL contributed to the interpretation of the results. All authors read, revised, and approved the final manuscript.

## Conflict of Interest Statement

The authors declare that the research was conducted in the absence of any commercial or financial relationships that could be construed as a potential conflict of interest.
