# Supplementary material for: Silencing of D-Lactate Dehydrogenase Impedes Glyoxalase System and Leads to Methylglyoxal Accumulation and Growth Inhibition in Rice
Source: Front Plant Sci. 2017 Dec 5;8:2071. doi: 10.3389/fpls.2017.02071 (PMC5723347; doi:10.3389/fpls.2017.02071)
Supplement: Supplementary file 1 [file Presentation_1.PDF]

Supplementary Material

Silencing of D-lactate dehydrogenase impedes glyoxalase system and leads to methylglyoxal accumulation and growth inhibition in rice

Baoguang An[1,2], Jie Lan[1], Xiaolong Deng[1], Silan Chen[2], Chao Ouyang[2], Huiyun Shi[1], Jing Yang[1] and Yangsheng Li[1]*

*** Correspondence:** Yangsheng Li: lysh2001@whu.edu.cn

1 Supplementary Figures and Tables

1.1 Supplementary Figures

```
EcD-LDH      1 -----MSSMTTTDNKAFLNELARLVGSSHLLTDPAKTARYRKGFRSGQGD
KlD-LDH      1 ------MFRFVGRSGFALRGSLQLRKDVLRSRTTAVAKRHYSSSNGNNGG
ScD-LDH      1 -----------------MWKRTCTRIKIARGRVRRSCYRYASTGTGSTDS
AtD-LDH      1 ----MAFASKFARSKTILSFLRPCRQLHSTPKSTGDVTVLSPVKGRRRLP
OsD-LDH-FL   1 MATAAAALLRLSRSRRPLLPLSSLRLPPPAPYHHHSHSQTPPSSSSSSSH
OsD-LDH-Tr   1 MATAAAALLRLSRSRRPLLPLSSLRLPPPAPYHHHSHSQTPPSSSSSSSH

EcD-LDH     46 ALAVVFPGSLLELWRVLKACVTADKIILMQAANTGLTEGSTPNGNDYDRD
KlD-LDH     45 GFSSAILSVLGGSLIGGGFVAYALG-SQFEKEKSVSDLSIARLEDLDSPE
ScD-LDH     34 SSWKYSVIASSATGYAKNYSRTKDIKMVKKIDVN----------------
AtD-LDH     47 TCWSSSLFPLAIAASATSFAYLNLSNPSISESSSALDSRDITVGGKDSTE
OsD-LDH-FL  51 ARLPAFLSFLAAAAAAAGGTTVALCDSGIDHR----------VGGKESTE
OsD-LDH-Tr  51 ARLPAFLSFLAAAAAAAGGTTVALCDSGIDHR----------VGGKESTE

EcD-LDH     96 VVIISTLRLDKLHVLGKGEQVLAYPGTTLYSLEKALKPLGREPHSVIG--
KlD-LDH     94 YCDKETFAKALVELKDVLENDPENFTVAKDDLDAHSDTYFNSHHAEANQR
ScD-LDH     68 -STKSSDSDYHDVKIDKVVDKVGNKNYSDAKSDDAHSDTYNTHH------
AtD-LDH     97 AVVKGEYKQVPKELISQLKTILEDNLTTDYDERYFHGKPQNSFHKAVN-I
OsD-LDH-FL  91 LVVRGERKRVPNEFIDELASFLGENLTVDYEERHYHGTPQNSFHKAVN-V
OsD-LDH-Tr  91 LVVRGERKRVPNEFIDELASFLGENLTVDYEERHYHGTPQNSFHKAVN-V

EcD-LDH    144 -------SSCIGASVIGGICNNSGGSLVQRGPAYTEMSLFARINEDGKLT
KlD-LDH    144 PEIVLYPRNTEDVSKLLKICHKYSIPVIPFSGGTSLEGHFLPTRPGSCVV
ScD-LDH    111 -----SRRIIHTTVSKIKICHDNNMVVSGGTSGHTRIGDTITVDS-----
AtD-LDH    146 PDVVVFPRSEEEVSKILKSCNEYKVPIVPYGGATSIEGHTLAPKGGVCID
OsD-LDH-FL 140 PDVVVFPRSQDEVQKIVMACNKYKVPIVPYGGATSIEGHTLAPHGGVCIN
OsD-LDH-Tr 140 PDVVVFPRSQDEVQKIVMACNKYKVPIVPYGGATSIEGHTLAPHGGVCIN

EcD-LDH    187 LVNHLG----IDLGETPEQILSKLDDDRIKDDDVRHDGRHAHDYDYVHRV
KlD-LDH    194 LDISKYLNKIIQLNKEDLDVVVQGGVPWEELNEYLNDHGLLFGCDPGPGA
ScD-LDH    151 --------KMNNVVKDKDDITVAG-----WDNDYSDHG---MGCDGG---
AtD-LDH    196 MSLMK---RVKALHVEDMDVIVEPGIGWLELNEYLEEYGLFFPLDPGPGA
OsD-LDH-FL 190 MSLMK---KIKSLHVEDMDVVVEPGVGWIELNEYLKPYGLFFPLDPGPGA
OsD-LDH-Tr 190 MSLMK---KIKSLHVEDMDVVVEPGVGWIELNEYLKPYGLFFPLDPGPGA

EcD-LDH    233 RDIEADTPARYNADPDRLFESSGCAGKLAVFAVRLDTFEAEKNQQVFYIG
KlD-LDH    244 QIAGCIANSCSGTNAYRYGTMKENVVNITMCMADGTIVKTKRRPRKSSAG
ScD-LDH    182 AIGGCIANSCSGTNAYRYGTMKN---IINMTIVDGTIVKTKKR-RKSSAG
AtD-LDH    243 SIGGMCATRCSGSLAVRYGTMRDNVISLKVVLPNGDVVKTASRARKSAAG
OsD-LDH-FL 237 TIGGMCATRCSGSLAVRYGTMRDNVINLQAVLPNGDVVKTGSRARKSAAG
OsD-LDH-Tr 237 TIGGMCATRCSGSLAVSL--------------------------------
```

```
EcD-LDH     283 TNQP------EVLTEIRRHILANFENLPVAGEYMHRDIYDIAEKYGKDTF
KlD-LDH     294 YNLNGLIIGSEGTLGIVTEATIKCHVRSTFETVAVVPFPTVSDAASCSSH
ScD-LDH     228 YNNG------VGSGTGIVTATVKCHVKKATVAVVSD---TIKDAAACASN
AtD-LDH     293 YDLTRLIIGSEGTLGVITEITLRLQKIPQHSVVAVCNFPTVKDAADVAIA
OsD-LDH-FL  287 YDLARLIIGSEGTLGVITEVTVRLQKLPSHSVVAMCNFQTIKDAADVAIA
OsD-LDH-Tr      --------------------------------------------------

EcD-LDH     327 LMIDKLGTDKMPFFFNLKGRTDAMLEKVKFFRPHFTDRAMQKFGHLFPSH
KlD-LDH     344 LIQAGIQLNAMELLDDNMMKIINQSGATSKDNWVESPTLFFKIGGRSEQI
ScD-LDH     269 TSG-------IHNAMDNMMKINASSTDR------CDWVKTMKIGG----R
AtD-LDH     343 TMMSGIQVSRVELLDEVQIRAINMANGK---NLTEAPTLMFEFIG-TEAY
OsD-LDH-FL  337 TMLSGIQVSRVELLDEVQIRAINMANGK---NLPEVPTLMFEFIG-TEAY
OsD-LDH-Tr      --------------------------------------------------

EcD-LDH     377 LPPRMKNWRDKYEHHLLLKMAGDGVGEAKSWLVDYFKQAEGDFFVCTPE-
KlD-LDH     394 IQEVIKEVEKIASQHNNTKFEFATDEDSKLELWEARKVALWSTIDTGRKT
ScD-LDH     302 SNIVNAVDVKAVANHCN------SAKDDDKWARKVRRWSVDADKSKD---
AtD-LDH     389 TREQTQIVQQIASKHNGSDFMFAEEPEAKKELWKIRKEALWACYAMA---
OsD-LDH-FL  383 ALEQTLLVQKIATEHHGSDFVFVEEPDAKEELWKIRKEALWAGFAMK---
OsD-LDH-Tr      --------------------------------------------------

EcD-LDH     426 -EGSKAFLHRFAAAGAAIRYQAVHSDEVEDILALDIALRRNDTEWYEHLP
KlD-LDH     444 NPDANIWTTDVAVPISKFADVINATKEEMNASGLLTSLVGHAGDGNFHAF
ScD-LDH     343 -KSAKIWTTDVAVVSDKVIHTKK------DMASKINAIVGHAGDG-NHAI
AtD-LDH     436 -PGHEAMITDVCVPLSHLAELISRSKKELDASSLLCTVIAHAGDGNFHTC
OsD-LDH-FL  430 -PDHEAMITDVCVPLSRLAECISVSKEKLDASPLTCLVIAHAGDGNFHTI
OsD-LDH-Tr      --------------------------------------------------

EcD-LDH     475 PEIDSQLVHKLYYGHFMCYVFHQDYIVKKGVDVHALKEQMLELLQQRGAQ
KlD-LDH     494 IIYN---TEQRKT---------AETIVENMVKRAIDAEGTCTGEHGVGIG
ScD-LDH     385 VYRTHTCSVDR--------------------MVKRANAGTCTG-HGVGIG
AtD-LDH     485 IMFDPSSEEQRRE---------AERLNHFMVHSALSMDGTCTGEHGVGTG
OsD-LDH-FL  479 ILFDPSQEDQRRE---------AERLNHFMVHTALSMEGTCTGEHGVGTG
OsD-LDH-Tr      --------------------------------------------------

EcD-LDH     525 YPAEHNVGHLYKAPETLQKFYRENDPTNSMNPGIGKTSKRKNWQEVE-
KlD-LDH     532 KRDYLLEQVGEDTVAVMRKLKLALDPKRILNPDKIFKIFKIDPNDHQH
ScD-LDH     414 KR-----------YGAVDMRKIKAIDKRIMNGN---------------
AtD-LDH     526 KMKYLEKELGIEALQTMKRIKKTLDPNDIMNPGKLIPPHVCF------
OsD-LDH-FL  520 KMKYLEKELGIESLRTMKRIKAALDPNNIMNPGKLIPPQVCI------
OsD-LDH-Tr      ------------------------------------------------
```

Figure S1. Alignment of OsD-LDH, EcD-LDH, KlD-LDH, ScD-LDH and AtD-LDH.

OsD-LDH shows high similarity with AtD-LDH. Black shaded boxes, identical amino acids; grey shaded boxes, similar amino acids. EcD-LDH, *Escherichia coli* D-lactate dehydrogenase; KlD-LDH, *Kluyveromyces lactis* D-lactate dehydrogenase; ScD-LDH, *Saccharomyces cerevisiae* D-lactate dehydrogenase; AtD-LDH, *Arabidopsis thaliana* D-lactate dehydrogenase/glycolate dehydrogenase; OsD-LDH-FL, the full-length rice D-Lactate dehydrogenase; OsD-LDH-Tr, the truncated rice D-Lactate dehydrogenase.

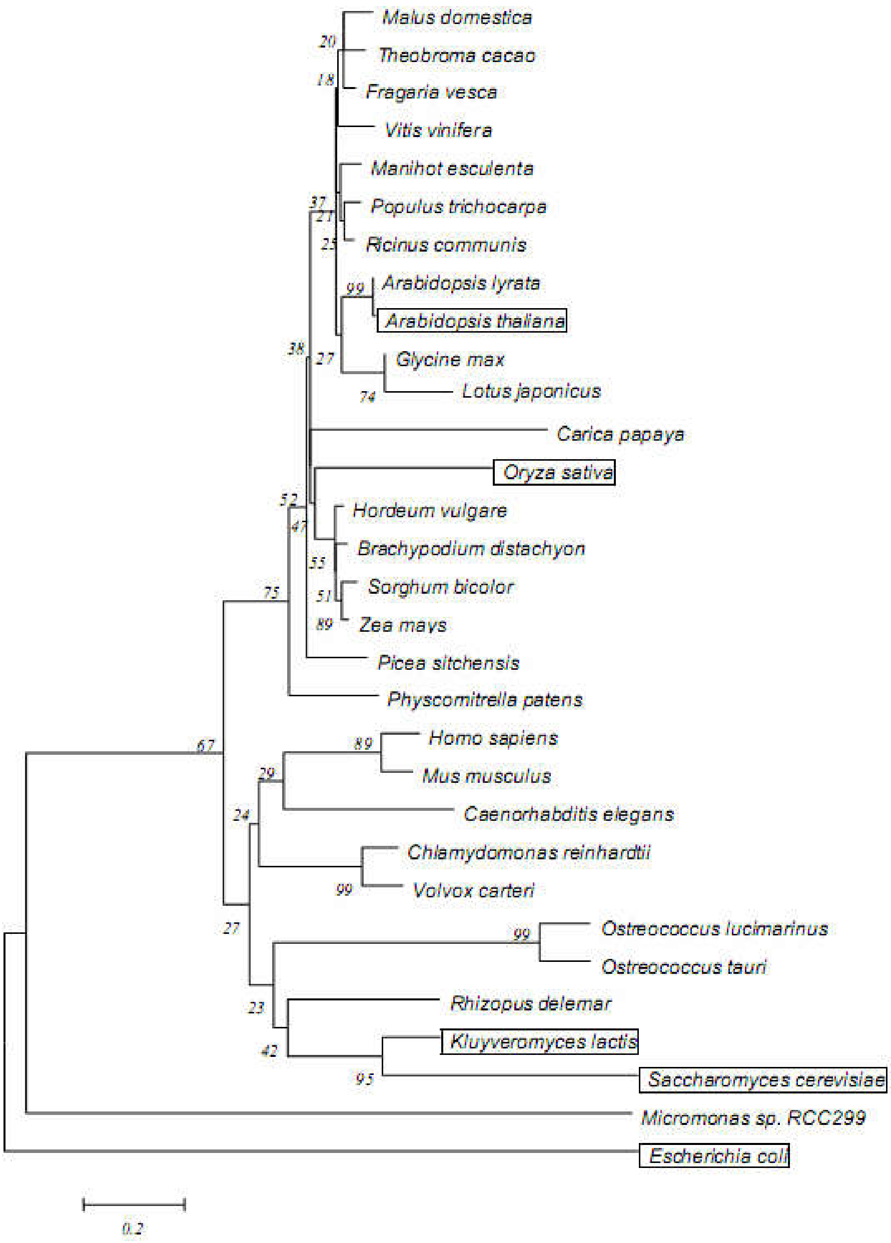

Figure S2. Phylogenetic tree of D-LDHs (EC 1.1.2.4).

The protein sequence of full-length OsD-LDH was used for a search for homologous genes using BLASTP algorithm. The obtained sequences were used to conduct a phylogenetic tree with MEGA5 using the neighbor-joining program with 1,000 bootstrap replications. D-LDHs that have been characterized were marked by pane. The PLAZA or GenBank accession numbers of the sequences were provided in Table S3.

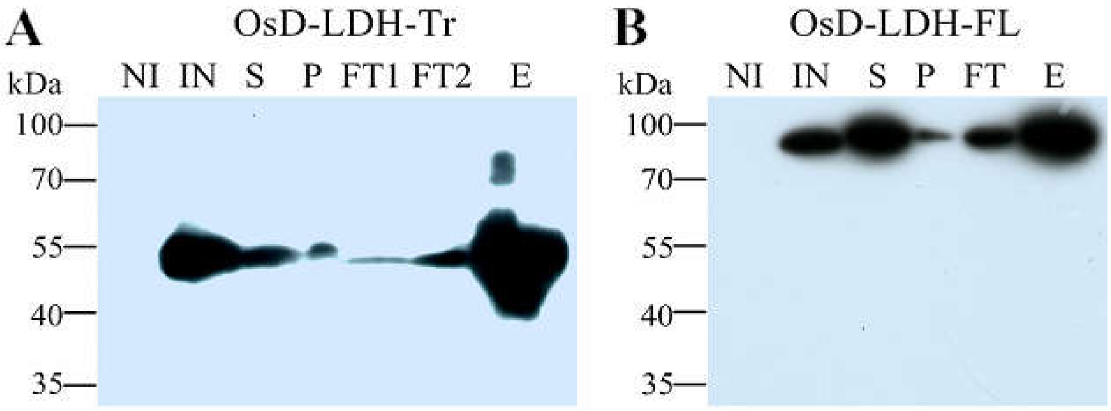

Figure S3. Identification of purified protein by Western blotting.

(A) and (B) Immunoblot of GST-OsD-LDH-Tr and GST-OsD-LDH-FL using anti-GST antibody, respectively. NI, Not induced cells; IN, Induced cells by IPTG; S, Supernant; P, Pellet; FT, Flow through; E, Elute protein; kDa, Kilodalton.

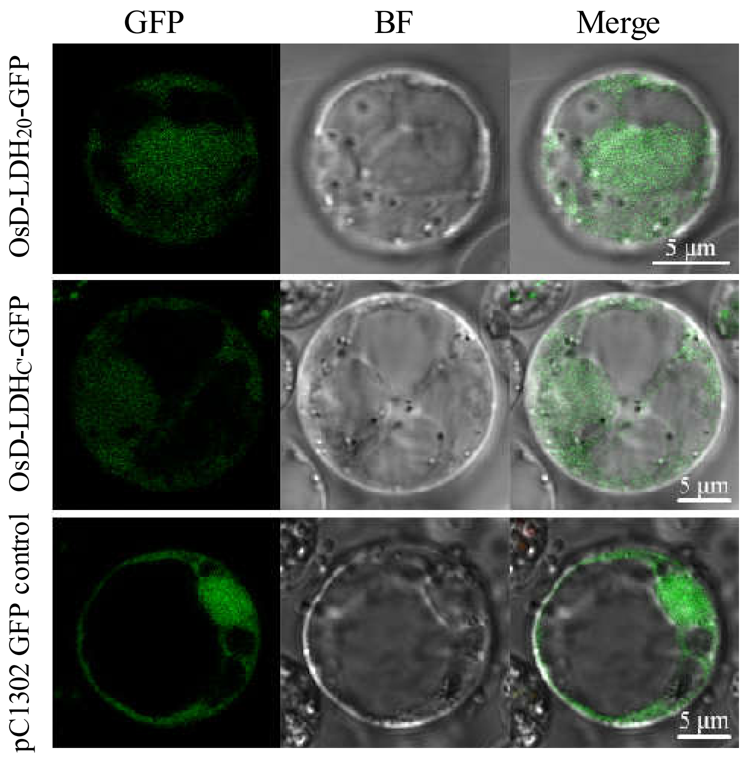

Figure S4. Subcellular localization of the signal peptides in protoplasts of transgenic rice plants.

OsD-LDH$_{20}$-GFP and OsD-LDH$_{C'}$-GFP, GFP fused to first N-terminal 20 amino acids of OsD-LDH and the C-terminus of full-length OsD-LDH (without the first 79 amino acids and the stop codon), respectively; pC1302 GFP control, Empty vector as the GFP control. All the signal peptides were fused in-frame to the N-terminus of GFPs. Mito-Red, mitochondrion specific dye Mito-Tracker Red; BF, Bright field. The bars were indicated in the figures.

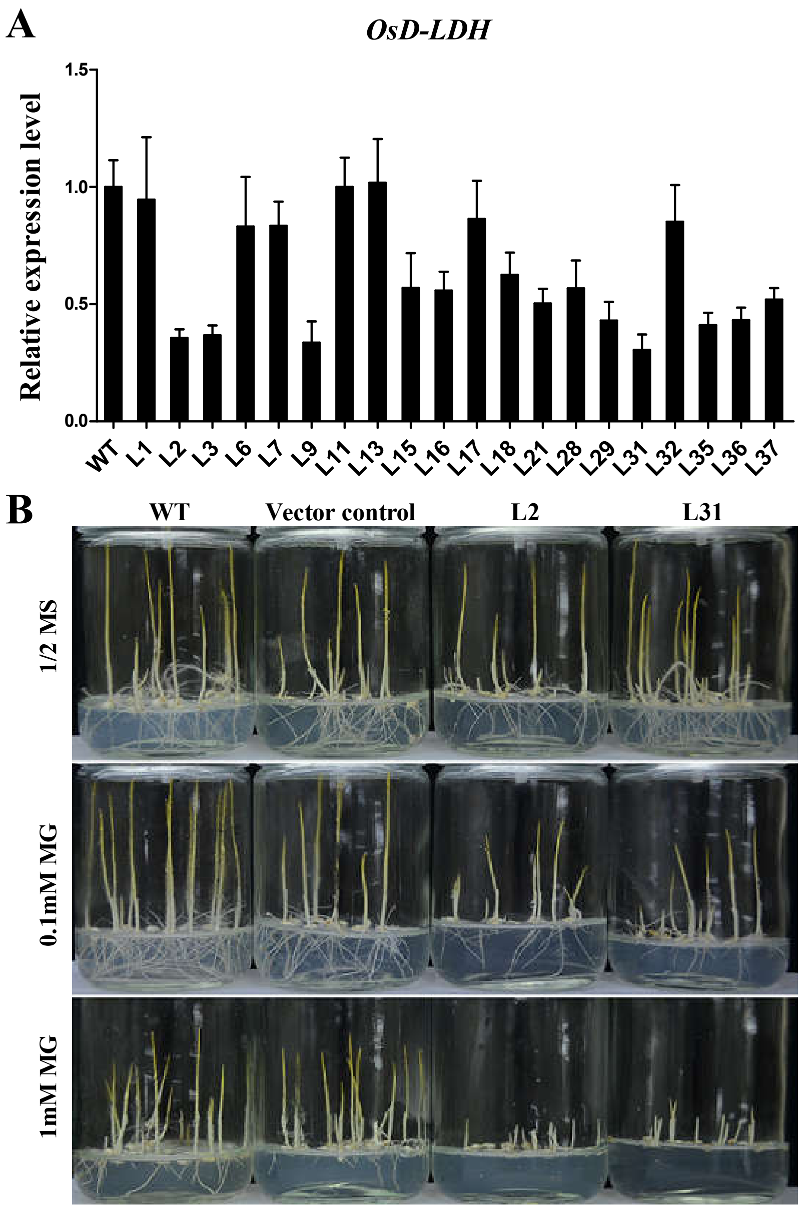

Figure S5. Expression of *OsD-LDH* in *OsD-LDH* RNAi transgenic plants and effect of methylglyoxal on 1-week-old seedlings.

(A) Two-week-old seedlings geminated on sterile water for wild type (WT) plants or selected with 30 mg L^{-1} hygromycin B for transgenic plants were used for detection of expression of *OsD-LDH*; Values represent the mean±SD (n=3). (B) One-week-old seedlings were grown on ½ MS solid media containing 2% sucrose for THE WT plants or selected with 30 mg L^{-1} hygromycin B for CK, *OsD-LDH* RNAi transgenic plants (L2 and L31). MG treatments were in the corresponding media

supplemented with different concentrations of MG, respectively. Three independent experiments with 15 seeds at least each time were performed.

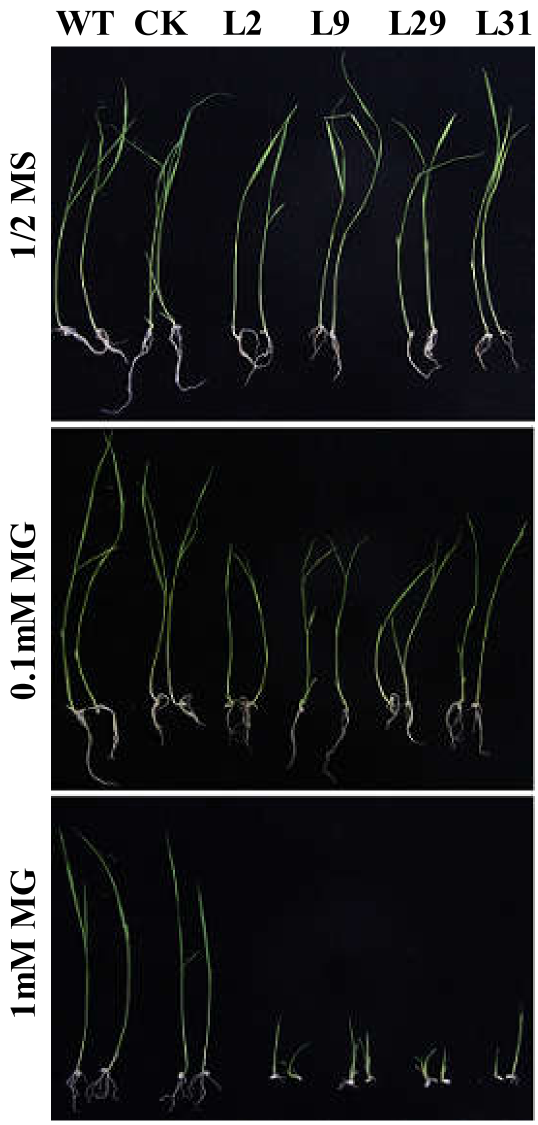

Figure S6. Effect of MG on 2-week-old seedlings eliminating the influence of sucrose.

Two-week-old seedlings were grown on the same media of Fig. 5 except that the media were not containing sucrose.

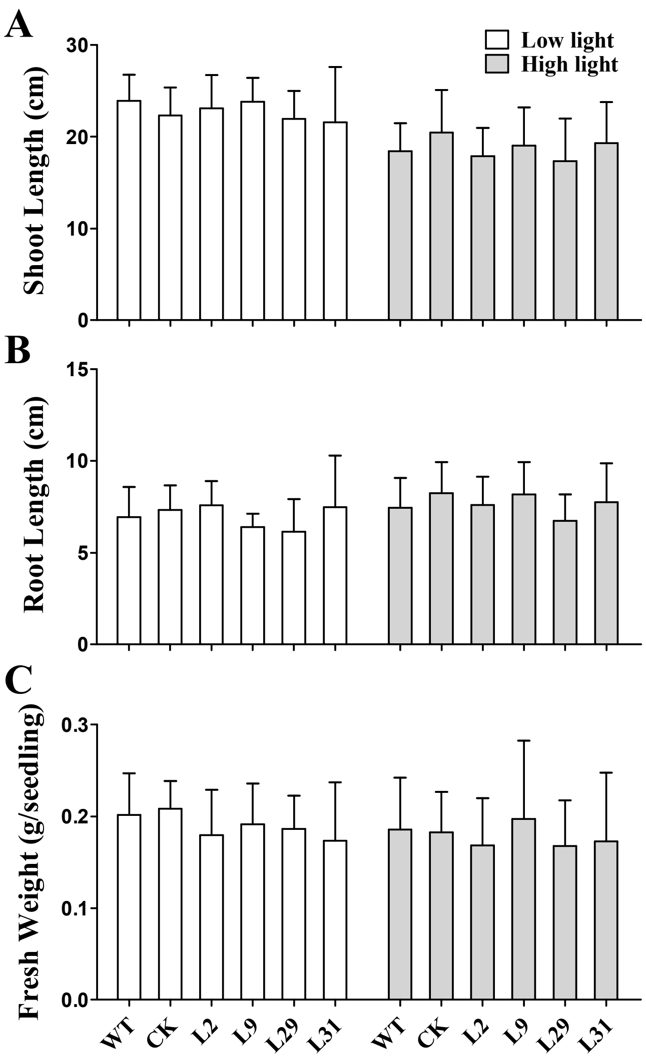

Figure S7. Growth status of 2-week-old seedlings grown under different light intensity.

(A-C) Statistic analyses of shoot length, root length and fresh weight of 2-week-old seedlings grown under different light intensity, respectively. Values represent the mean ± SD (n=30). Low light, light intensity was 120 μmol photons m^{-2} s^{-1}; High light, light intensity was 1000μmol photons m^{-2} s^{-1}. WT, wild type plants; CK, empty vector control; L2, L9, L29 and L31, *OsD-LDH* RNAi plants.

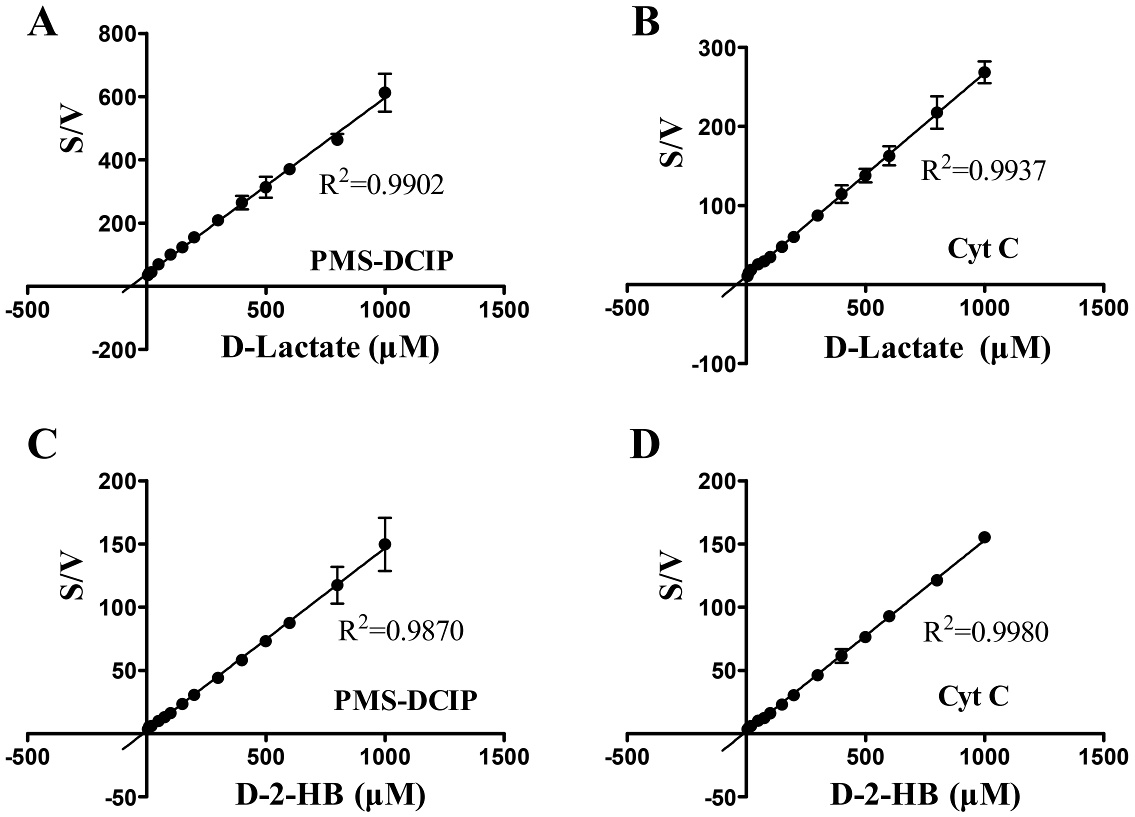

Figure S8. Kinetic parameters of OsD-LDH were measured using a Hanes–Woolf plot. (A-D) Kinetic parameters of OsD-LDH toward D-lactate and D-2-HB using PMS-DCIP and Cyt c as electron acceptors, respectively; V_{max} and K_M were calculated using a Hanes–Woolf plot method and all experiments were performed at 30°C and pH 8.35. D-2-HB, D-2-hydroxybutyrate; Values represent the mean ± SD (n=3).

1.2 Supplementary Tables

Table S1. Alternative splicing variants of LOC_Os07g06890

Alt 3′SS, Alternative 3′ splice site. * number represented the number of changed base pairs.

AS type	AS location	Change on CDS sequence (5’--3’)	Basic types of	Change on protein sequence	Number of clones	Frequency of

	on CDS	(Insertion▼) (DeletionΔ)	AS			occurrence
I				Encoding 254 aa, truncated protein	24	51.1%
II	759	(ΔTTTGTAG)7*	Alt 3'SS	Encoding 561 aa, full-length protein	23	48.9%

Table S2. Alternative splicing of D-*LDH* in other species

Species	ESTs Locus	
	with "TTTGTAG" like Type I	without"TTTGTAG" like Type II
Aquilegia	DR943529	DT747637
Capsicum annuum	GD127748	
Carica papaya	EX274772	EX265038
Zea mays	EE175024,CO440348	EE032563,DT941837,etc.

Table S3. Accession numbers of D-LDH sequences in the PLAZA or GenBank database used in the phylogenetic analysis

Species	Accession no.
Malus domestica	MD12G004100
Theobroma cacao	TC05G020040
Fragaria vesca	FV6G28180
Vitis vinifera	VV08G01030

Manihot esculenta	ME03777G00060
Populus trichocarpa	PT06G19790
Ricinus communis	RC29333G00590
Arabidopsis lyrata	AL6G06240
Arabidopsis thaliana	AT5G06580
Glycine max	GM03G36390
Carica papaya	CP00222G00080
Lotus japonicus	LJ0G051490
Hordeum vulgare	Bradi1g73170
Glycine max	BAJ97979
Oryza sativa	OS07G06890
Brachypodium distachyon	BD1G56880
Sorghum bicolor	SB02G003640
Zea mays	ZM02G46640
Picea sitchensis	ABK24997
Physcomitrella patens	PP00018G00430
Homo sapiens	NP_705690
Mus musculus	NP_081846
Chlamydomonas reinhardtii	CR10G03720

Volvox carteri	VC00044G00280
Medicago truncatula	Medtr8g146560
Caenorhabditis elegans	NP_001023872
Ostreococcus lucimarinus	OL08G03420
Ostreococcus tauri	OT08G03390
Rhizopus delemar	EIE80616
Kluyveromyces lactis	CAA50635
Saccharomyces cerevisiae	CAA46852
Escherichia coli	CAA25531
Micromonas sp. RCC299	MRCC299_03G01560

Table S4. Subcellular localization of glyoxalases predicted by TagertP

OsGlyI-6.1 and OsGlyI-6.2 represented proteins translated from different alternative splicing variants and other descriptions were similar. cTP, chloroplast target peptide; mTP, mitochondrial target peptide; SP, secretory pathway signal peptide; other, some other place of the cell; TP length, length of target peptide; C, M and S in Location column represent localization in chloroplast, mitochondrion and secretory proteins, respectively.

Protein Name	Locus	cTP score	mTP score	SP score	other score	Location	TP length
GlyI-1	LOC_Os01g0785	0.314	0.350	0.035	0.169	M	39

	0.1						
GlyI-2	LOC_Os02g17920.1	0.417	0.469	0.036	0.003	M	61
GlyI-3	LOC_Os03g16940.1	0.559	0.171	0.046	0.416	C	17
GlyI-4	LOC_Os03g45720.1	0.049	0.431	0.018	0.814	—	—
GlyI-5	LOC_Os04g45590.1	0.242	0.057	0.110	0.599	—	—
OsGlyI-6.1	LOC_Os05g07940.1	0.481	0.330	0.037	0.099	C	29
OsGlyI-6.2	LOC_Os05g07940.2	0.042	0.314	0.390	0.159	S	17
OsGlyI-6.3	LOC_Os05g07940.3	0.510	0.343	0.049	0.088	C	29
OsGlyI-6.4	LOC_Os05g07940.4	0.289	0.476	0.066	0.145	M	40
OsGlyI-6.5	LOC_Os05g07940.5	0.121	0.079	0.055	0.955	—	—
OsGlyI-7.1	LOC_Os05g14194.1	0.530	0.770	0.017	0.003	M	48

OsGlyI-7.2	LOC_Os05g14194.2	0.530	0.770	0.017	0.003	M	48
OsGlyI-8	LOC_Os05g22970.1	0.847	0.263	0.115	0.003	C	46
OsGlyI-9.1	LOC_Os07g06660.1	0.959	0.119	0.013	0.031	C	55
OsGlyI-9.2	LOC_Os07g06660.2	0.959	0.119	0.013	0.031	C	55
OsGlyI-10	LOC_Os07g46360.1	0.039	0.380	0.074	0.835	—	—
OsGlyI-11.1	LOC_Os08g09250.1	0.185	0.083	0.113	0.870	—	—
OsGlyI-11.2	LOC_Os08g09250.2	0.185	0.083	0.113	0.870	—	—
OsGlyI-11.3	LOC_Os08g09250.3	0.185	0.083	0.113	0.870	—	—
OsGLYII-1.1	LOC_Os01g47690.1	0.600	0.422	0.008	0.005	C	47
OsGLYII-1.2	LOC_Os01g47690.2	0.600	0.422	0.008	0.005	C	47
OsGLYII-2	LOC_Os03g2146	0.125	0.107	0.404	0.455	—	—

17

	0.1					
OsGLYII-3	LOC_Os09g3410<br>0.1	0.631	0.277	0.047	0.015	C	17
